# Internal dynamics and guest binding of a sterically overcrowded host†

**DOI:** 10.1039/c6sc00985a

**Published:** 2016-04-04

**Authors:** Susanne Löffler, Jens Lübben, Axel Wuttke, Ricardo A. Mata, Michael John, Birger Dittrich, Guido H. Clever

**Affiliations:** a Faculty for Chemistry and Chemical Biology, TU Dortmund University Otto-Hahn-Str. 6 44227 Dortmund Germany guido.clever@tu-dortmund.de; b Institute for Inorganic Chemistry, Georg-August-University Göttingen Tammannstr. 4 37077 Göttingen Germany; c Institute for Physical Chemistry, Georg-August-University Göttingen Tammannstr. 6 37077 Göttingen Germany; d Institute for Inorganic Chemistry and Structural Chemistry, Heinrich-Heine University Düsseldorf Universitätsstr. 1 40225 Düsseldorf Germany

## Abstract

Substituent control in self-assembled host systems allows for a fine-tuning of structure, dynamics and guest preference. Flat banana-shaped ligands L^1^ assemble with Pd(ii) cations into the interpenetrated coordination cage dimer [3BF_4_@Pd_4_L^1^_8_], capable of sequential guest uptake. In contrast, the introduction of bulky adamantyl groups in ligand L^2^ prevents dimerization and results in the clean formation of monomeric cage species [Pd_2_L^2^_4_]. Owing to steric crowding, the adamantyl substituent is considerably bent sideways with respect to the ligand backbone, and is rapidly flipping between both faces of the free ligand giving rise to two energetically degenerate conformers. Surprisingly, the flipping is preserved in the cage, albeit at a lower rate due to entropic reasons. Despite the very dense packing within the self-assembled structure, the cage is able to encapsulate a series of bis-anionic guests in an induced-fit fashion. Electronic structure calculations revealed a substantial contribution from dispersion interactions between the guest and the surrounding adamantyl groups that stabilize the host–guest complex. Guest exchange kinetics were quantified and the influence that encapsulated guests imparted on the ligand flipping dynamics was examined by a series of 2D NMR experiments. Four synchrotron X-ray structures of the cage and its host–guest complexes are presented, allowing for unprecedented insight into the host–guest interactions of a sterically overcrowded host and its guest-induced distortion.

## Abstract 2

Despite its cramped interior, a [Pd_2_L_4_] coordination cage consisting of bulky adamantyl ligands shows impressive motility and a rich encapsulation chemistry.

## Graphical abstract

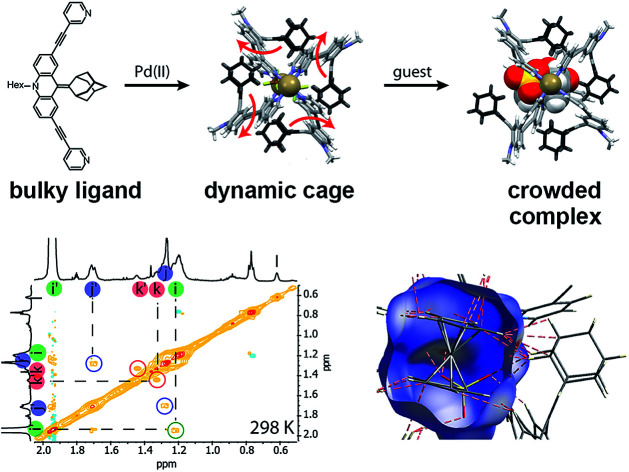

## Introduction

Dynamic processes play a central role in the vast majority of biomolecular systems. Proteins can change their conformation upon substrate binding, DNA is actively unwound for transcription and various molecular motors are able to transform chemical into mechanical energy.^1^ In order to gain a better understanding of such dynamic processes in complex molecular systems, and furthermore, to utilize these principles in future applications, supramolecular chemists have been studying artificial systems that implement selected dynamic features for the last couple of decades. Examples include light-controllable shuttling^1,2^ and rocking^3^ motions in rotaxanes, pirouetting motion of rings in catenanes,^4^ supramolecular ball bearings,^5^ cog wheels,^6^ propellers,^7^ invertible helices,^8^ lockable gates,^9^ scissors,^10^ caterpillars,^11^ molecular valves,^12^ light-powered engines,^13,14^ and unidirectional walkers.^15,16^ Some of these systems combine dynamic behaviour with molecular-scale cavities such as the rotaxane–MOF hybrid systems reported by Loeb and coworkers^17,18^ and the flexible porous framework materials introduced by Kitagawa,^19^ Fischer,^20^ Garcia-Garibay and others.^21^

In the field of metallo-supramolecular self-assembly of discrete rings and cages,^22^ several examples of dynamic functionality fused to rigid architectures in exohedral positions have been developed by Yang^23^ and Stang.^23,24^ Other self-assembled structures were designed containing flexible functionalities as integral parts of their framework. Noteworthy is the tetrahedral cage consisting of six rotaxane edges that has been recently reported by Nitschke, Sanders and Schalley.^25^ A further degree of complexity is achieved in cages with inbuilt switching mechanisms, controlled by external stimuli such as light. Fujita and coworkers have reported a spherical cage in which the photoisomerization of endohedrally arranged azobenzenes modifies the hydrophobicity of the cavity environment.^26^ In contrast, Zhou have reported a coordination cage containing azobenzene substituents in exohedral positions, in which the photoswitching affects supramolecular aggregation processes.^27^ We have recently introduced self-assembled coordination cages based on dithienylethene (DTE) photoswitches in which the inherent flexibility of the ligand backbones can be switched by light with drastic effects on guest binding and assembly size.^28^ Dynamic host–guest systems have not only been examined with respect to guest affinity and uptake/release kinetics, but also regarding the motility of guests inside the cavity in terms of tumbling or flipping motions.

For example, Raymond and coworkers have studied the dynamics of various benzyl phosphonium guest derivatives when encapsulated in self-assembled tetrahedral cages.^29^ The group of Schmittel has investigated the tumbling of a DABCO molecule inside a supramolecular cavity flanked with two Zn-porphyrin complexes.^7^ Similar effects have also been observed in purely organic supramolecular container molecules.^30^ The processes observed in these non-covalent host–guest systems share similarities with molecular gyroscopes in which a central, dynamic element is covalently bound to a surrounding cage or ring structure.^31–33^ Steric interactions between the guest and surrounding host structure, as well as the associated solvent molecules, have been identified as important factors that modulate the dynamics of such systems by what may be called molecular friction.^31^

Several examples of self-assembled cages have been reported, in which steric bulk was systematically incorporated into the framework in order to modulate the guest binding properties. Severin and coworkers have recently reported a family of voluminous ditopic ligands featuring an iron-clathrochelate core that assemble into sterically congested [Re_4_L_4_] rings and [Pd_6_L_12_] cages.^34,35^ Yoshizawa has employed a range of metal-mediated and organic capsules using large anthracene panels and studied their aggregation and guest binding properties.^36^ We have installed steric bulk into the backbone of bis-monodentate ligands to control the dimerization and guest binding selectivity of interpenetrated double cages.^37^ Furthermore, the Fujita group has shown that also remotely installed steric bulk can influence the uptake of guests inside self-assembled cages.^38^

The direct consequence of introducing steric bulk into a host system is the reduction of the size of the internal cavity and the width of the portals that allow for entry and release of guest molecules, thereby restricting the size of the encapsulated guest and slowing down their exchange kinetics.^39^ However, the common belief that steric crowding is always repulsive in nature (and thus generally detrimental to guest binding) seems to be wrong. Even in the absence of functional groups that supposedly dominate intermolecular interactions such as hydrogen bond donors or acceptors, attractive interactions between closely associated molecules are always promoted by London dispersion interactions.^40^ Though individually weak, dispersive forces can add up substantially in larger systems such as supramolecular assemblies.^41^ Although dispersion interactions have been known since the 1930's, this insight has only recently become actively discussed again by the broader community, as evidenced by coining the definition of “dispersion energy donors” (DEDs) and the emphasis given to the explicit treatment of dispersion in modern methods of electronic structure calculation.^42^ This motivated us to synthesize self-assembled host systems equipped with bulky but otherwise unfunctionalized groups in order to study the interplay between molecular crowding, self-assembly and host–guest chemistry.

## Results and discussion

### Cage and guest design

**Fig. 1:**
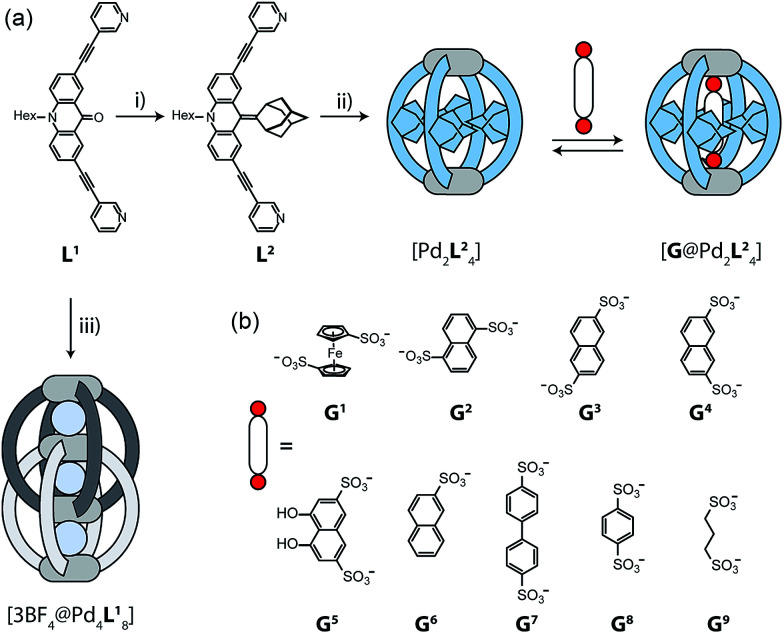
(a) Synthesis of ligand L^2^ and assembly to cage [Pd_2_L^2^_4_]; (i) adamantone, TiCl_3_, LiAlH_4_, NEt_3_, THF; (ii) [Pd(CH_3_CN)_4_](BF_4_)_2_, CD_3_CN, 70 °C, 15 min (in contrast, ligand L^1^ assembles to the interpenetrated double-cage [3BF_4_@Pd_4_L^1^_8_]; (iii) [Pd(CH_3_CN)_4_](BF_4_)_2_, CD_3_CN, 70 °C, 12 h). Addition of a guest G to [Pd_2_L^2^_4_] leads to the formation of a host–guest complex [G@Pd_2_L^2^_4_]. (b) Structures of various guest molecules encapsulated by coordination cage [Pd_2_L^2^_4_].

The design of our system is based on a banana-shaped bis-monodentate pyridyl ligand with a tricyclic acridone backbone.^43^ Previously, we reported the synthesis and self-assembly of acridone ligand L^1^ with Pd(ii) cations yielding interpenetrated double cages [3BF_4_@Pd_4_L^1^_8_], a mechanically interlocked dimer of two [Pd_2_L^1^_4_] cages that contains tetrafluoroborate anions in its three pockets (Fig. 1, lower left).^44^ We further showed that the outer two BF_4_^−^ ions can be replaced by chloride or bromide anions *via* an allosteric binding mechanism with positive cooperativity, thereby rendering the central pocket susceptible for the uptake of small neutral guest molecules such as benzene or cyclohexane. Here, we report the functionalization of this ligand by replacing the carbonyl substituent with a doubly-bound adamantylidene group, thereby dramatically increasing steric bulk in the centre of the ligand L^2^ (Fig. 1a). We investigate this ligand's capability of forming [Pd_2_L^2^_4_] self-assembled cages in which the adamantyl groups serve as dispersion energy donors contributing to the binding of guests inside the cage's cavity. In addition, cationic palladium centres are arranged at opposite ends across the globular cavity. As shown before, rod-shaped bis-anionic guests carrying a negative charge at either end are encapsulated in such [Pd_2_L_4_] cages in a way that the guest's major axis is collinear with the Pd–Pd axis, thereby bringing the anionic centres as close as possible to the metal cations.^45^ In order to probe the interactions within the sterically crowded equatorial area of cage [Pd_2_L^2^_4_], we focus on studying the uptake of various bis-anionic guests with respect to their relative *lateral* bulk. Therefore, this study complements our previous work where we systematically studied the guest binding affinity in a related (but not as bulky) [Pd_2_L_4_] cage as a function of the *lengths* of a series of bis-anionic guests, resulting in a system resembling a molecular ruler.^46^

### Ligand synthesis and cage assembly

**Fig. 2:**
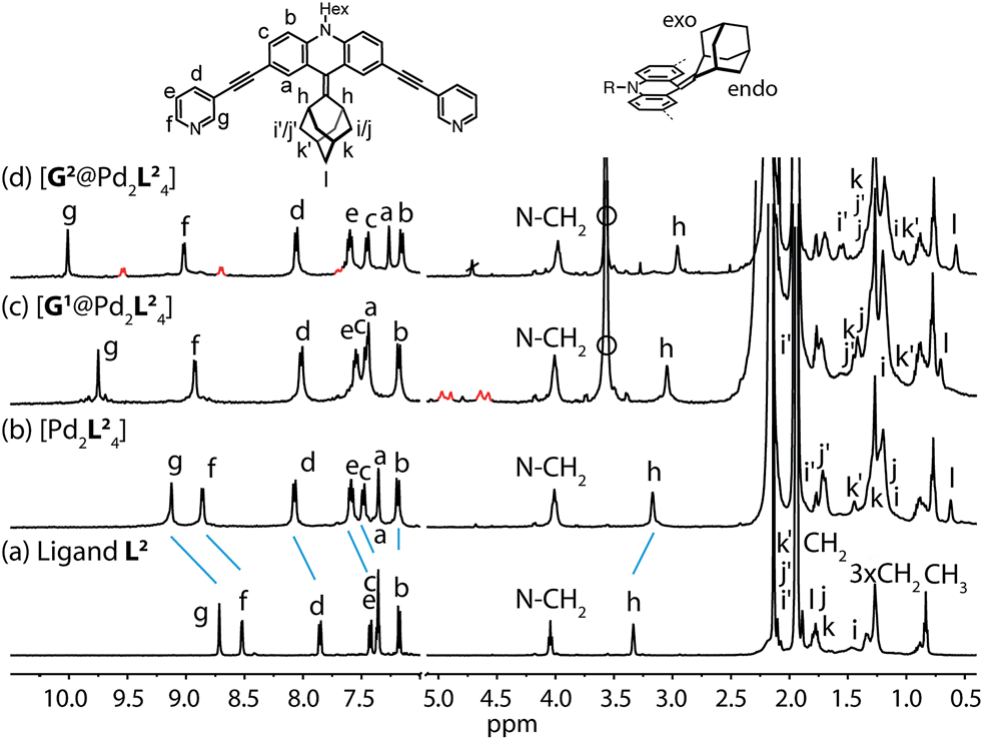
^1^H NMR spectra of the cage and some host–guest complexes. (a) Ligand L^2^, (b) cage [Pd_2_L^2^_4_], (c) [G^1^@Pd_2_L^2^_4_], and (d) [G^2^@Pd_2_L^2^_4_] (400 MHz, 298 K, CD_3_CN). The signals of the encapsulated guest molecules are highlighted in red. H_i_, H_j_ and H_k_ represent outside (*exo*) and H_i′_, H_j′_ and H_k′_ inside (*endo*) pointing hydrogen atoms of the adamantyl substituent with respect to the acridone backbone (compare Fig. 4a). Empty circle: [K(18-crown-6)]^+^.

Starting from the previously reported ligand L^1^ the new ligand L^2^ was synthesized *via* a McMurry coupling with adamantone using titanium(iii)-chloride and lithium aluminium hydride, affording the desired product in 85% yield. The ^1^H NMR spectrum of the ligand revealed the adamantyl group gave rise to two sets of signals (*exo* and *endo* with respect to the ligand backbone, see Fig. 4a), indicating a bent shape of the ligand with the adamantyl substituent flipped to one side (Fig. 2a). Heating ligand L^2^ for 15 min at 70 °C in the presence of 0.5 equivalents of [Pd(CH_3_CN)_4_](BF_4_)_2_ in deuterated acetonitrile resulted in the quantitative formation of the monomeric cage [Pd_2_L^2^_4_], as indicated by the shifting of all ^1^H NMR signals (Fig. 2b). The pyridine signals shifted downfield, which indicates the coordination of the nitrogen donors to the palladium(ii) cations, while the adamantyl signals shifted upfield due to the close proximity of the adamantyl substituents to the aromatic backbones of the neighbouring ligands. Dimerization into an interpenetrated double cage, however, was not observed.

**Fig. 3:**
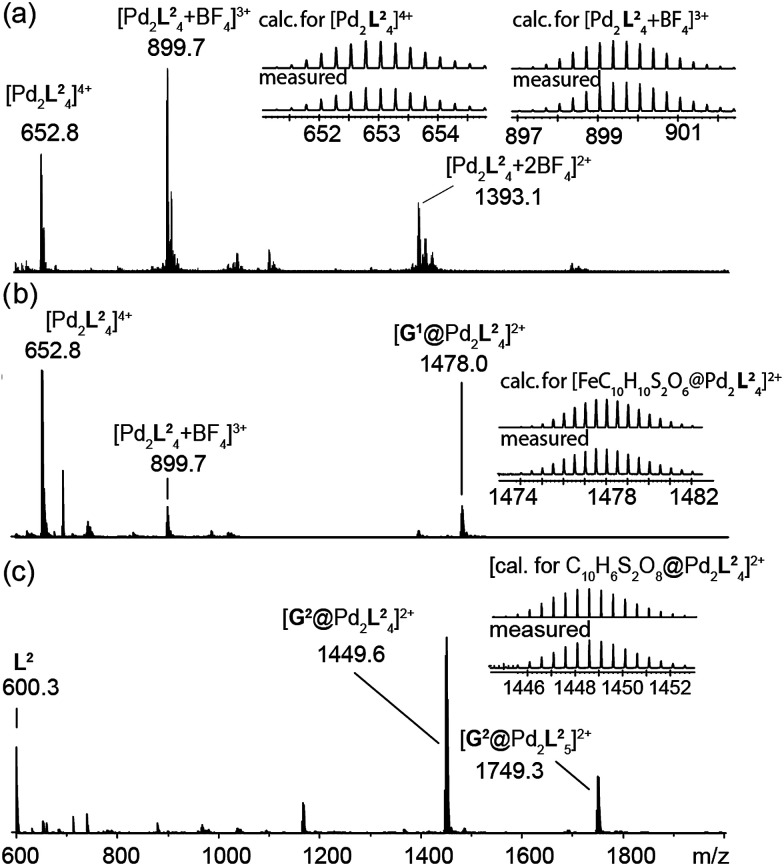
ESI mass spectra of the cage and some host–guest complexes. (a) [Pd_2_L^2^_4_], (b) [G^1^@Pd_2_L^2^_4_], and (c) [G^2^@Pd_2_L^2^_4_].

The high-resolution ESI mass spectrum of the self-assembly product clearly supports the formation of the cage [Pd_2_L^2^_4_] by exhibiting a series of species [Pd_2_L^2^_4_ + *n*BF_4_]^(4−*n*)+^ (*n* = 0–2) containing a variable number of BF_4_^−^ counter anions (Fig. 3a).

### Dynamics

The attachment of the adamantyl residue to the central sp^2^-hybridized carbon on the concave side of the ligand has interesting structural consequences: in contrast to the flat backbone of L^1^, ligand L^2^ is bent with the adamantyl residue flipped to one side. This is caused by the close contact between the hydrogen atoms attached to the double-bond's next neighbours in the backbone and the adamantyl substituent.

**Fig. 4:**
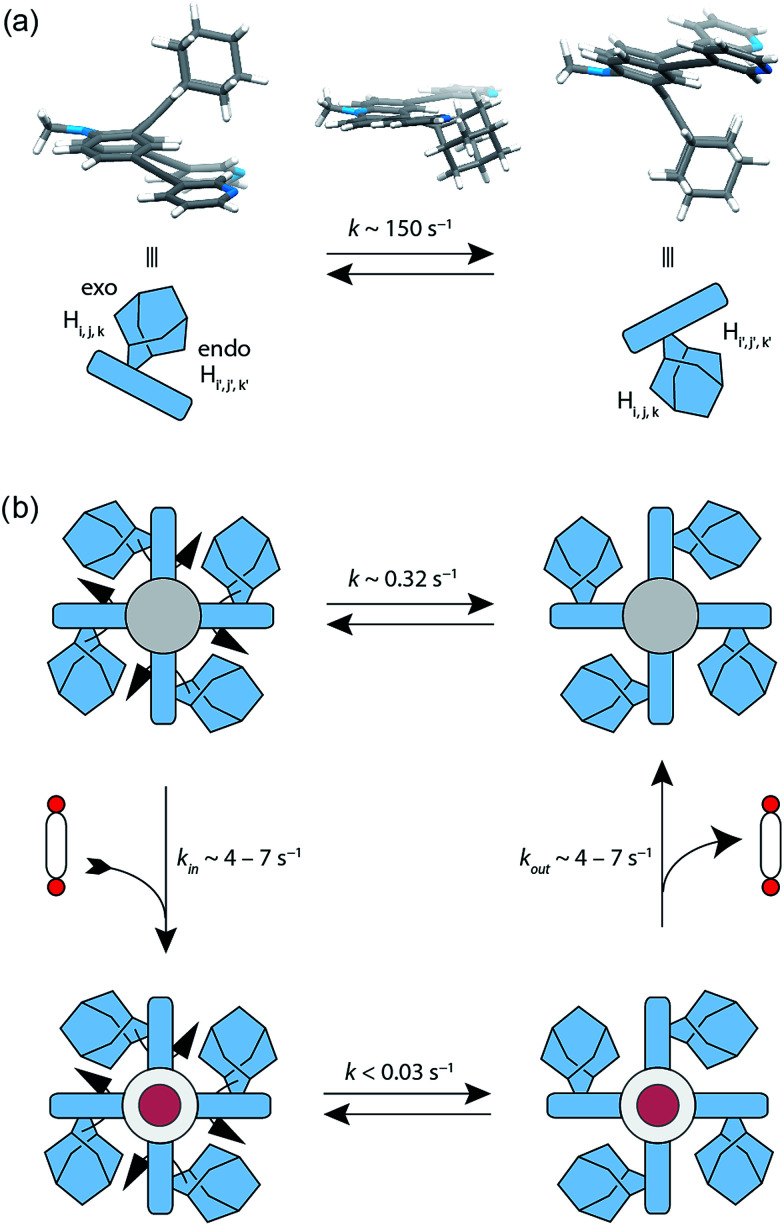
Flipping rates in ligand L^2^, coordination cage [Pd_2_L^2^_4_] and host–guest complexes [G^1^@Pd_2_L^2^_4_] and [G^2^@Pd_2_L^2^_4_]. (a) Depiction of the degenerate minimum conformations of ligand L^2^ and the calculated transition state. The flipping rate constant of the free ligand L^2^ was experimentally determined to *k* ∼ 150 s^−1^ at 298 K. (b) The flipping rate in the ‘empty’ cage [Pd_2_L^2^_4_] was determined to *k* ∼ 0.32 s^−1^. After addition of bis-anionic guests G^1^ or G^2^, the rate constant decreased to *k* < 0.03 s^−1^.

^1^H–^1^H NOESY NMR experiments on ligand L^2^ show correlations between the *exo* (H_i_, H_j_ and H_k_) and the *endo* (H_i′_, H_j′_ and H_k′_) protons of the adamantyl group (Fig. SI 16†). The corresponding cross-peaks intensify with increasing temperature, hence indicating a dynamic movement of the adamantyl group within the ligand structure. As rotation of the adamantyl group can be excluded, due to the presence of the double bond, we propose that a flipping motion occurs in the ligand that allows the *endo* and *exo* faces of the adamantyl substituent to swap positions (Fig. 2 and 4a). The rate constant for the flipping was determined from the 2D NMR experiment to be *k* ≈ 150 s^−1^ at 298 K (for comparison, all rate constants were determined at the same temperature of 298 K). A comparable case of dynamic exchange between two degenerate conformations was previously described for a structurally related dicyanomethylene-substituted acridone, although the electronic situation in this push–pull system differs considerably from electron-rich ligand L^2^.^47^

The formation of the monomeric cage [Pd_2_L^2^_4_] was expected to have a tremendous effect on the motility of the adamantyl groups. To our surprise, the flipping dynamics of the adamantyl groups can still be observed in the self-assembled cage, albeit at much lower rates. VT (variable temperature) ^1^H–^1^H NOESY NMR experiments were performed for cage [Pd_2_L^2^_4_] in order to obtain the flipping rates at different temperatures, from which the activation parameters were calculated by the Eyring plot method (ESI†). The flipping process inside the cage is slowed down significantly with respect to the free ligand, with a rate constant of *k* ≈ 0.32 s^−1^ (Fig. 4b, SI 17 and SI 18†). This is mainly caused by an unfavourable entropic contribution (Δ*S*^‡^_ligand_ = 11.4 J mol^−1^ K^−1^*vs.* Δ*S*^‡^_cage_ = −164.5 J mol^−1^ K^−1^). The slower flipping can be explained by the high degree of steric crowding inside the supramolecular self-assembly. Interestingly, from an enthalpic point of view, the flipping inside the cage is favoured over flipping in the free ligand (Δ*H*^‡^_ligand_ = 64.0 kJ mol^−1^*vs.* Δ*H*^‡^_cage_ = 26.9 kJ mol^−1^). A computational study on the free ligand supported the proposed flipping motion: at ambient temperatures (298 K) the activation barrier was calculated to be Δ*G*^‡^_298,calc_ = 56.6 kJ mol^−1^, comparing well with the experimental value of Δ*G*_298,exp_ = 60.6 kJ mol^−1^ extracted from the NMR data. The calculated structures of the conformational minimum and the transition state of the flipping motion are depicted in Fig. 4a (for further details see the Computational section).

To explain the mechanism of the adamantyl flipping motion within the densely packed coordination cage, four possible mechanisms were proposed and investigated: (a) full decomplexation of one ligand, subsequent flipping of all adamantyl groups in the tentative, sterically relaxed [Pd_2_L^2^_3_] fragment, followed by recomplexation; (b) one-side decomplexation of one ligand, then flipping of all four adamantyl groups and recomplexation; (c) concerted flipping of all adamantyl groups passing each other simultaneously in the centre of the cage and (d) sequential flipping of each adamantyl group of the intact cage. A series of experiments was conducted in order to deduce the most plausible mechanism. A ^1^H EXSY NMR measurement of a 3 : 1 mixture of free ligand L^2^ and the [Pd_2_L^2^_4_] cage showed no exchange between those two species on the NMR time scale (Fig. SI 25†). Therefore, a mechanism following a complete dissociation of one of the ligands (suggestion a) could be excluded. A subsequent experiment showed that the addition of up to five equivalents of pyridine as a competitive ligand to the [Pd_2_L^2^_4_] cage does not lead to any significant changes in the rate constant of the flipping motion (Fig. SI 26†). If one-side dissociation of a ligand would be involved in the cage's flipping mechanism, the addition of a competing ligand should increase the flipping rate, since additional donors can be used to accelerate ligand exchange of square-planar metal centres that proceed through an associative mechanism.^48^ As the pyridine–platinum bond is kinetically much more robust than the palladium analogue,^49^ also a platinum-based cage was prepared by heating ligand L^2^ with [Pt_2_Cl_2_(CH_3_CN)_2_] and AgClO_4_ for 3 days at 80 °C. Again, ^1^H–^1^H NOESY NMR experiments on cage [Pt_2_L^2^_4_] show flipping of the adamantyl groups inside the cage, with an exchange rate (*k* ≈ 0.13 s^−1^) similar in magnitude to that of the palladium cage (Fig. SI 19†).

Both of the latter two experiments rule out that a partial dissociation pathway is involved in the flipping mechanism (suggestion b). Furthermore, the concerted flipping of all adamantyl groups (suggestion c) is very unlikely for steric reasons (there is not enough space in the interior of the cage to harbour the four adamantyl groups). Therefore, we hypothesize that the flipping is a sequential movement (suggestion d).

### Host–guest chemistry

**Fig. 5:**
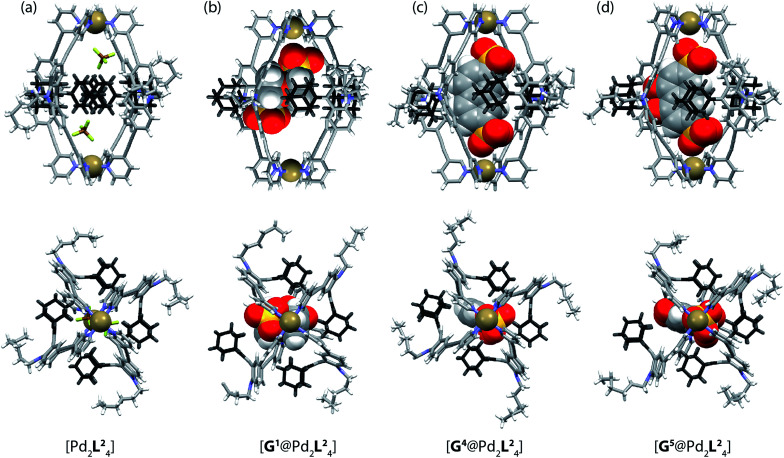
X-ray crystal structures of (a) [Pd_2_L^2^_4_], (b) [G^1^@Pd_2_L^2^_4_], (c) [G^4^@Pd_2_L^2^_4_] and (d) [G^5^@Pd_2_L^2^_4_]. Color scheme: C, grey; N, blue; O, red; S, yellow; F, green; B, brown; Pd, tan; Fe, orange. For clarity, solvent molecules and free (non encapsulated) anions are omitted. The ESI and CCDC numbers 1053080–83 contain details of the crystallographic data.

To investigate the host–guest chemistry of the sterically crowded cage, a number of mono- and bis-anionic guests G (G^1^–G^9^) were titrated into a solution of the cage [Pd_2_L^2^_4_] in acetonitrile (see Fig. 1b for the structures of the guest molecules). It was initially expected that the tremendous steric bulk of the four equatorial adamantyl groups would only allow for guests with a thin backbone (*i.e.* an alkyl chain) to bind inside the cage. However, we were surprised to see that even quite bulky guests are bound to form host–guest complexes [G@Pd_2_L^2^_4_]. This was verified by NMR spectroscopy (Fig. 2c and d and ESI†), high-resolution mass spectrometry (Fig. 3) and single crystal X-ray analysis (Fig. 5, see discussion below). Depending on the size and charge of the examined guest molecule, a different binding behaviour was observed. After addition of the bis-anionic guests G^1^–G^5^ to the [Pd_2_L^2^_4_] cage a new set of signals emerged in the ^1^H NMR spectra, corresponding to the formed host–guest complexes. For example, addition of 1,1′-ferrocene bis(sulfonate) G^1^ or 2,6-naphthalene bis(sulfonate) G^2^ to cage [Pd_2_L^2^_4_] leads to a noticeable change of the chemical shift of the inward pointing protons (H_g_, H_i′_, H_j′_ and H_k′_), indicating encapsulation of the guest molecules (Fig. 2c and d). The ^1^H NMR signals of the encapsulated guests were found to be relatively sharp and shifted downfield. The high-resolution mass spectra showed signals assignable to the host–guest systems [G^1^@Pd_2_L^2^_4_]^2+^ and [G^2^@Pd_2_L^2^_4_]^2+^, respectively, whose experimental isotopic patterns were in agreement with the calculated peak distributions (Fig. 3b and c).

A case worth discussing in more detail is the encapsulation of the 1,1′-ferrocene bis(sulfonate) guest G^1^. The ^1^H NMR spectrum of the host–guest complex (Fig. 2c) revealed that the two signals, assigned to the cyclopentadienyl protons in α and β position relative to the sulfonate substituent, are further split twofold upon encapsulation by the host.^50^ This splitting pattern arises from the desymmetrization of the *C*_2h_-symmetric ferrocene derivative (*anti* conformation) once transferred into the *C*_4h_-symmetric coordination cage. The number of the cage's NMR signals, however, does not change, indicating that the guest is rapidly rotating inside the cavity. In accordance with our previous studies, cyclic voltammetry showed that the Fe(ii)/Fe(iii) redox-potential is anodically shifted upon encapsulation of the ferrocene inside the cationic cage (Fig. SI 27†). This can be explained by the effect of the tetracationic cage disfavouring the removal of an electron from the encapsulated ferrocene guest.^45^

Due to the substantial size of the guests, these are in close contact with the surrounding adamantyl residues in the equatorial region of the cavity as can be observed in the X-ray structures (Fig. 5) and also in the NOESY NMR spectra recorded for the host–guest complexes. Fig. SI 22 in the ESI† highlights close contacts between the cage's adamantyl residues and the protons of guest G^2^. After one equivalent of the guests G^1^–G^5^ has been added to the cage, the signals of the empty [Pd_2_L^2^_4_] cage disappeared and only signals of the host–guest systems [G^1–5^@Pd_2_L^2^_4_] remained in the ^1^H NMR spectra. Hence, guests G^1^–G^5^ are strongly bound inside the cage (with association constants estimated to be larger than 10^4^ to 10^5^ L mol^−1^) and the guest exchange with the solvent exterior is slow on the NMR time scale. In contrast, addition of guests G^6^–G^9^ to cage [Pd_2_L^2^_4_] resulted in a gradual shifting of the cage's ^1^H NMR signals, indicating a fast exchange of these guests. Since more than one equivalent of guests G^6^–G^9^ was required to saturate the cages, these guests bind more weakly to the cage than those previously discussed.^51^ The different binding behaviour is a result of differences in the guest structures. Guest molecules G^1^–G^5^ are bis-anionic and have a good size match for encapsulation,^46^ while the other guests are either too small (G^8^ and G^9^), too large (G^7^) or lack a second sulfonate group (G^6^) to be encapsulated with high affinity inside the coordination cage. An NMR exchange spectroscopy (EXSY) experiment of a 50 : 50 sample of empty cage [Pd_2_L^2^_4_] and host–guest complex [G^1^@Pd_2_L^2^_4_] or [G^2^@Pd_2_L^2^_4_] was employed to determine the exchange rates of the guests as *k* ≈ 7 s^−1^ for [G^1^@Pd_2_L^2^_4_] and *k* ≈ 4 s^−1^ for [G^2^@Pd_2_L^2^_4_] (Fig. 4 and ESI Fig. SI 23 and SI 24†). In comparison, the previously reported monomeric cage composed of a dibenzocycloheptatriene-based ligand L^3^, which is of similar length and backbone structure but does not have a bulky adamantyl group, shows an exchange rate of *k* ≈ 90 s^−1^ with the bis(sulfonate) guest G^1^ (Fig. SI 15†).^52^ This led us to conclude that the bulky adamantyl groups of ligand L^2^ congest the portals of cage [Pd_2_L^2^_4_] and slow down the rate of guest exchange. As we discuss below, we hypothesize that they also contribute to the thermodynamic stabilization of the guest molecules inside the cavity.

### Guest effect on the flipping dynamics

Next, we investigated the effect of encapsulation of the bis(sulfonate) guests G on the flipping rates of the adamantyl groups in the host–guest complexes [G^1^@Pd_2_L^2^_4_] and [G^2^@Pd_2_L^2^_4_] by high-resolution ^1^H NMR spectroscopy (900 MHz). For the following experiments, [K(18-crown-6)]^+^ was used as counter cation for the guests since the ^1^H NMR signals of the NBu_4_^+^ species overlapped with relevant ligand signals. The rate constants of the adamantyl flipping in the host–guest systems [G^1^@Pd_2_L^2^_4_] and [G^2^@Pd_2_L^2^_4_] were determined to be *k* < 0.03 s^−1^ and *k* < 0.02 s^−1^, respectively. The flipping is more than 10 times slower than in cage [Pd_2_L^2^_4_] containing only tetrafluoroborate counter anions (Fig. 4b). The exchange rates of guests G^1^ and G^2^ were determined to be *k* = 7 s^−1^ and *k* = 4 s^−1^, respectively. This is several hundred times faster than the flipping motion. Taken together, we anticipate that the guests leave the cage while the adamantyl groups change their position.

### X-ray structure analyses

Single crystals of cage [Pd_2_L^2^_4_] suitable for X-ray structure determination were grown by slow vapour diffusion of diethyl ether into an acetonitrile solution of the cage. Diffraction data for this and the other crystals was collected at the SLS synchrotron source. The X-ray crystal structure reveals a *C*_4h_-symmetric [Pd_2_L^2^_4_] cage, occupied by two BF_4_^−^ anions that are positioned near the Pd(pyridine)_4_-planes (Fig. 5a). The distance between the two Pd atoms was measured to be 16.20 Å. The ligand shows the distinctive bent shape, arising from the heavy deformation of the tricyclic acridone backbone, in which the central ring adopts a boat conformation.

The double bond between the backbone and the adamantyl substituent itself is not significantly bent, with all six carbon atoms constituting the tetrasubstituted double bond system occupying a common plane. All adamantyl groups are bowed sideways with radial symmetry and occupy the four portals of the cage. This results in a highly packed structure with short inter-ligand distances, measuring only 3.11 Å from proton H_a_ to proton H_j′_ of the neighbouring ligand. The cavity provides a rather spacious environment near the Pd(pyridine)_4_-planes (where the anions are found), while the bulky adamantyl groups reach into the centre of the cage leaving a narrow channel measuring about 6.3 Å in diameter (see the ESI† for further views of the X-ray structures). In addition, single crystals of the host–guest systems [G^1^@Pd_2_L^2^_4_], [G^4^@Pd_2_L^2^_4_] and [G^5^@Pd_2_L^2^_4_] were obtained *via* slow vapour diffusion of methanol (for [G^1^@Pd_2_L^2^_4_]), benzene (for [G^4^@Pd_2_L^2^_4_]) and diethyl ether (for [G^5^@Pd_2_L^2^_4_]) into acetonitrile solutions of these complexes.

In all three cases, the relatively large guest molecules were encapsulated inside the cages, with each guest orienting its anionic substituents in close proximity to the Pd(ii) cations. This is most pronounced for guests G^4^ and G^5^ but less so for shorter ferrocene-based guest G^1^ that is only able to closely approach one of the palladium atoms (Fig. 5). Interestingly, guest encapsulation forces the adamantyl groups of the ligands to bend even further towards the outside of the cage (Fig. 5b–d). According to DFT computational results, this guest-induced overstretching of the ligand should not cost an energetic penalty of more than 4 kJ mol^−1^.

Table 1 summarizes a selection of distances extracted from the X-ray structures of the cage and its host–guest complexes. An interesting trend can be observed in this data: the more bulky guests push the adamantyl residues further away from the cavity, which in turn leads to a gradual shortening of the cage along the Pd–Pd axis. In this sense, the cage behaves similar to a flexible container that slightly shrinks from top to the bottom when the cargo is so bulky that it bulges out the vessel to the sides.

**Table 1:** Selected distances extracted from the X-ray structures of [Pd_2_L^2^_4_] and some of its host–guest complexes

	[2BF_4_@Pd_2_L^2^_4_]	[G^4^@Pd_2_L^2^_4_]	[G^5^@Pd_2_L^2^_4_]	[G^1^@Pd_2_L^2^_4_]
Mol. volume of the guest [Å^3^]^a^	109.7	226.2	239.0	256.1
Distance [Å] between adamantyl sp^2^-C atom and center of cage^b^	**5.4** c	**6.1**	**6.9**	**6.8**
	5.3	5.5	5.5	5.8
	5.2	5.0	5.4	5.7
	5.2	5.0	5.1	5.6
Average distance [Å]	5.3	5.4	5.7	6.0
Pd–Pd distance [Å]	16.2	16.2	15.9	16.0

a Calculated from the DFT (B3LYP/6-31G*)^53^ structure of the anions.

b As defined by the middle of the line connecting the two palladium atoms.

c The longest distance each is highlighted in bold font.

### Analysis of non-covalent interactions

**Fig. 6:**
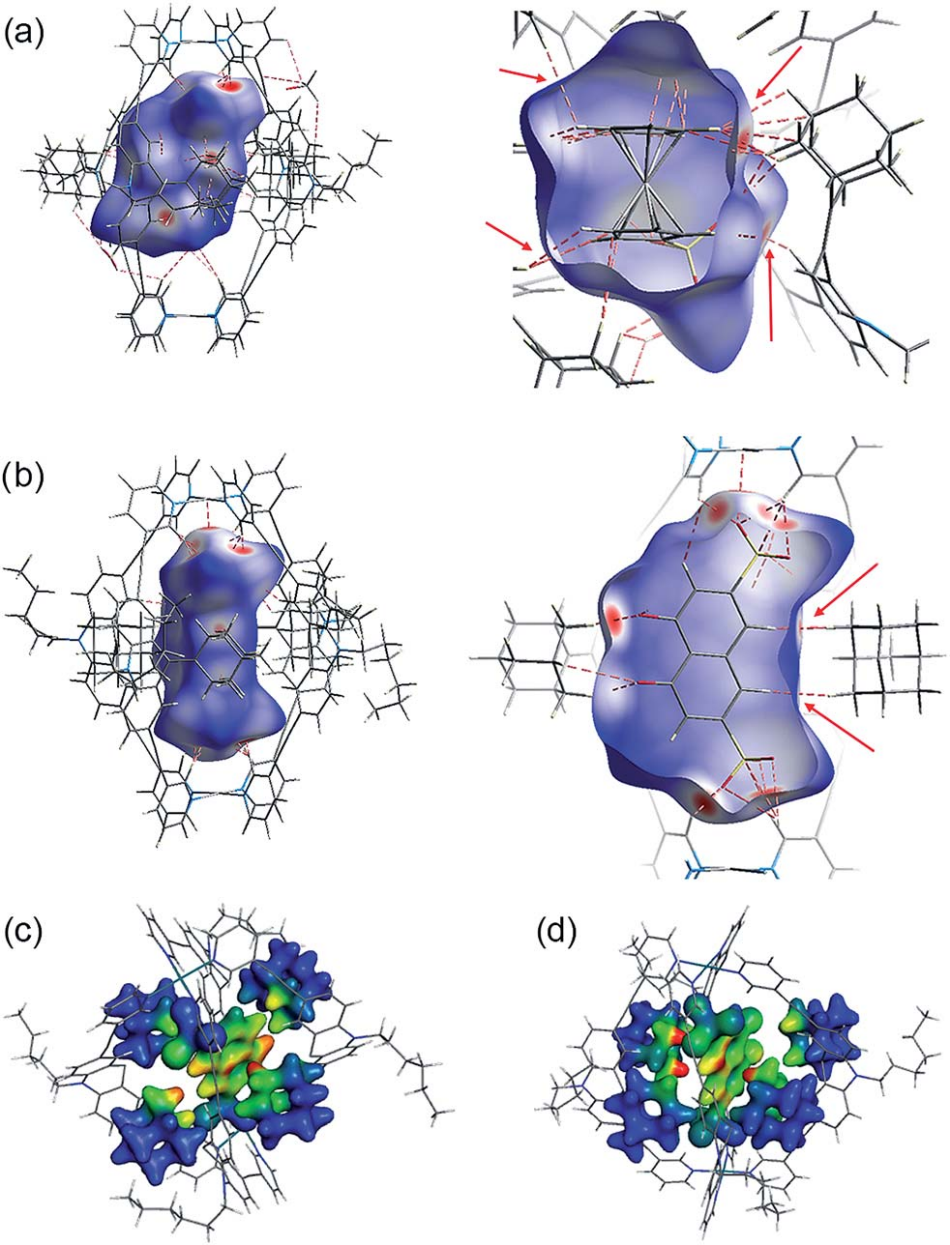
Visualization of non-covalent host–guest interaction. Hirshfeld *d*_norm_ surfaces for the guest molecules in (a) [G^1^@Pd_2_L^2^_4_] and (b) [G^5^@Pd_2_L^2^_4_] (plotted with isovalues from −0.4 (red: short contact) to 1.4 (blue: long contact); red dotted lines and arrows indicate some close non-hydrogen bond interactions that are shorter than 3.0 Å). (Below) Depiction of dispersion interaction densities (DIDs) between the guests and the surrounding adamantyl groups in (c) [G^4^@Pd_2_L^2^_4_] and (d) [G^5^@Pd_2_L^2^_4_] (red: high DID; blue: low DID).

Several short interactions varying from 2.0–3.0 Å between the cage and the encapsulated guest were observed in the X-ray structures. In order to more systematically visualize the non-covalent contacts in the host–guest assemblies, we performed a Hirshfeld surface analysis using the software Crystal Explorer.^54^ As depicted in Fig. 6a and b, the Hirshfeld surfaces were mapped with a van der Waals-radius-normalized distance *d*_norm_ around the guest molecules for host–guest complexes [G^1^@Pd_2_L^2^_4_] and [G^5^@Pd_2_L^2^_4_], respectively. Red areas highlight close contacts that contribute to the stabilization of the inclusion compound. Unsurprisingly, major contacts were found between the sulfonate oxygen atoms and the inward pointing pyridine hydrogen atoms. These hydrogens are polarized by the pyridines' involvement in the metal coordination and therefore readily available for serving as hydrogen bond donors (which is also expressed by the changes in their ^1^H NMR chemical shifts upon guest binding). In addition, also a number of close contacts were observed between the guests' hydrogen substituents or π-faces and the surrounding adamantyl hydrogens, characteristic of the dense packing inside the sterically congested cage. Element-filtered surface pictures and fingerprint plots are given in the ESI† to further visualize the range of non-covalent interactions at the interface of the guest and the surrounding host.

Electronic structure calculations were then carried out in order to obtain further insight into the binding of the guest molecules, in particular the role of dispersion forces. The parent cage containing only BF_4_^−^ counter anions as well as two host–guest systems were considered, namely [G^4^@Pd_2_L^2^_4_] and [G^5^@Pd_2_L^2^_4_]. First, the contribution of dispersion interactions to the cage stability was computed. Calculations at the SCS-LMP2/aug′-cc-pVTZ level of theory^55^ gave a dispersion contribution of 14.3 kJ mol^−1^ for the [Pd_2_L^2^_4_] system. The dispersion contribution in the [G^4^@Pd_2_L^2^_4_] system was calculated to be 9.3 kJ mol^−1^, somewhat smaller given the larger distances between the four ligands that result from the guest-induced expansion of the cage structure. This is not a particularly large effect and although it will counterbalance the steric repulsion of the bulky adamantyl moieties, it should not be a major factor in the cage formation itself.

Interestingly, dispersion forces seem to play a significant role in binding of the guest molecules inside the cages interior. The overall interaction energies between the adamantyl residues and the guests (excluding the cation–anion interactions) for the systems [G^4^@Pd_2_L^2^_4_] and [G^5^@Pd_2_L^2^_4_] were computed as 52.8 kJ mol^−1^ and 67.2 kJ mol^−1^. The dispersion energy contributions obtained are 86.3 kJ mol^−1^ and 64.0 kJ mol^−1^ for [G^4^@Pd_2_L^2^_4_] and [G^5^@Pd_2_L^2^_4_], respectively. These correspond to a strong contribution in binding, even exceeding the total interaction in the case of [G^4^@Pd_2_L^2^_4_]. In [G^5^@Pd_2_L^2^_4_], the adamantyl units are further apart, reducing the relative weight of the dispersion forces. Consequently, we anticipate the main contributions for the adamantyls' interaction with the guests to be Pauli repulsion and dispersion, the two of opposite sign. These results clearly show that dispersion forces can easily add up to large values in such a supramolecular construct. To better visualize this effect, we have plotted the Dispersion Interaction Densities (DIDs), calculated between the guest and the four adamantane units (Fig. 6c and d; the definition of DID is provided in the ESI†). The images show that the π-systems of the guests interact strongly with the surface of the pocket which is in accordance with the results of the Hirshfeld analysis. In particular, some hot spots are identifiable where the adamantyl groups are in close contact with the encapsulated guests.

## Conclusions

In this paper, we presented a bis-monodentate pyridyl ligand L^2^ that carries a bulky adamantyl group protruding sidewards from its concave face. The ligand cleanly assembled into monomeric *C*_4h_-symmetric coordination cages [Pd_2_L^2^_4_] or [Pt_2_L^2^_4_] in which the adamantyl groups occupy the four portals of the cage and part of the internal cavity. Rapid flipping of the adamantyl substituent was observed in the free ligand but dynamics were found to be drastically slowed down in the cage. Since the latter process did not require ligand detachment, we postulate that flipping of the adamantyls inside the cage proceeds *via* a sequential mechanism. The cage is able to encapsulate a range of bis(sulfonate) guest molecules G^1^–G^9^ in its interior. Consequently, the flipping motion of the ligand was further decelerated in the host–guest complexes [G@Pd_2_L^2^_4_]. Single crystal X-ray structures of the free cage and three host–guest complexes were obtained showing that the larger guests squeeze into the cavity by pushing the adamantyl residues further aside. This in turn leads to a slight compression of the cage along its Pd–Pd-axis. We investigated the non-covalent contacts between the guests and the surrounding host and identified a substantial contribution of attractive dispersion interactions conveyed by the adamantyl groups. Currently, we are extending the scope of dispersion energy donors (DEDs) that can be implemented into the ligand backbones. Since both dynamic scaffolds and densely packed molecular surroundings play important roles in the chemical processes taking place inside biological cavities (such as enzyme pockets), we believe that the type of artificial self-assemblies described herein will assist in understanding the interplay between molecular crowding and reactivity. In addition, we anticipate that such sterically overcrowded host structures in which the guests are snapping into a narrow inner cavity contribute new ideas to the field of supramolecular catalysis.^56^

## Supplementary Material

SC-007-C6SC00985A-s001

SC-007-C6SC00985A-s002

## References

[cit1] Lodish H. , Berk A., Kaiser C. A., Krieger M., Scott M. P., Bretscher A., Ploegh H. and Matsudaira P. T., Molecular Cell Biology, W. H. Freeman & Co Ltd, 6th edn, 2007

[cit2] Coskun A., Friedman D. C., Li H., Patel K., Khatib H. A., Stoddart J. F. (2009). J. Am. Chem. Soc..

[cit3] Hirose K., Ishibashi K., Shiba Y., Doi Y., Tobe Y. (2008). Chem.–Eur. J..

[cit4] Létinois-Halbes U., Hanss D., Beierle J. M., Collin J.-P., Sauvage J.-P. (2005). Org. Lett..

[cit5] Hiraoka S., Hirata K., Shionoya M. (2004). Angew. Chem., Int. Ed..

[cit6] Stevens A. M., Richards C. J. (1997). Tetrahedron Lett..

[cit7] Samanta S. K., Samanta D., Bats J. W., Schmittel M. (2011). J. Org. Chem..

[cit8] Miyake H., Kamon H., Miyahara I., Sugimoto H., Tsukube H. (2008). J. Am. Chem. Soc..

[cit9] Guenet A., Graf E., Kyritsakas N., Allouche L., Hosseini M. W. (2007). Chem. Commun..

[cit10] Muraoka T., Kinbara K., Aida T. (2006). Nature.

[cit11] Liu S., Kondratuk D. V., Rousseaux S. A. L., Gil-Ramírez G., O'Sullivan M. C., Cremers J., Claridge T. D. W., Anderson H. L. (2015). Angew. Chem., Int. Ed..

[cit12] Nguyen T. D., Tseng H. R., Celestre P. C., Flood A. H., Liu Y., Stoddart J. F., Zink J. I. (2005). Proc. Natl. Acad. Sci. U. S. A..

[cit13] Balzani V., Clemente-Leon M., Credi A., Ferrer B., Venturi M., Flood A. H., Stoddart J. F. (2006). Proc. Natl. Acad. Sci. U. S. A..

[cit14] Koumura N., Zijlstra R. W., van Delden R. A., Harada N., Feringa B. L. (1999). Nature.

[cit15] Delius von M., Geertsema E. M., Leigh D. A. (2010). Nat. Chem..

[cit16] Balzani V., Credi A., Raymo F., Stoddart J. (2000). Angew. Chem., Int. Ed..

[cit17] Loeb S. J. (2005). Chem. Commun..

[cit18] Vukotic V. N., Harris K. J., Zhu K., Schurko R. W., Loeb S. J. (2012). Nat. Chem..

[cit19] Kitagawa S., Uemura K. (2005). Chem. Soc. Rev..

[cit20] Schneemann A., Bon V., Schwedler I., Senkovska I., Kaskel S., Fischer R. A. (2014). Chem. Soc. Rev..

[cit21] Lin Z.-J., Lu J., Hong M., Cao R. (2014). Chem. Soc. Rev..

[cit22] Chakrabarty R., Mukherjee P. S., Stang P. J. (2011). Chem. Rev..

[cit23] Yang H.-B., Ghosh K., Northrop B. H., Zheng Y.-R., Lyndon M. M., Muddiman D. C., Stang P. J. (2007). J. Am. Chem. Soc..

[cit24] Yan X., Cook T. R., Pollock J. B., Wei P., Zhang Y., Yu Y., Huang F., Stang P. J. (2014). J. Am. Chem. Soc..

[cit25] Black S. P., Stefankiewicz A. R., Smulders M. M. J., Sattler D., Schalley C. A., Nitschke J. R., Sanders J. K. M. (2013). Angew. Chem., Int. Ed..

[cit26] Murase T., Sato S., Fujita M. (2007). Angew. Chem., Int. Ed..

[cit27] Park J., Sun L.-B., Chen Y.-P., Perry Z., Zhou H.-C. (2014). Angew. Chem., Int. Ed..

[cit28] Han M., Michel R., He B., Chen Y.-S., Stalke D., John M., Clever G. H. (2013). Angew. Chem., Int. Ed..

[cit29] Mugridge J. S., Szigethy G., Bergman R. G., Raymond K. N. (2010). J. Am. Chem. Soc..

[cit30] Harthong S., Dubessy B., Vachon J., Aronica C., Mulatier J.-C., Dutasta J.-P. (2010). J. Am. Chem. Soc..

[cit31] Kottas G., Clarke L., Horinek D., Michl J. (2005). Chem. Rev..

[cit32] Skopek K., Hershberger M., Gladysz J. (2007). Coord. Chem. Rev..

[cit33] Setaka W., Koyama A., Yamaguchi K. (2013). Org. Lett..

[cit34] Wise M. D., Ruggi A., Pascu M., Scopelliti R., Severin K. (2013). Chem. Sci..

[cit35] Wise M. D., Holstein J. J., Pattison P., Besnard C., Solari E., Scopelliti R., Bricogne G., Severin K. (2014). Chem. Sci..

[cit36] Yoshizawa M., Klosterman J. K. (2014). Chem. Soc. Rev..

[cit37] Freye S., Michel R., Stalke D., Pawliczek M., Frauendorf H., Clever G. H. (2013). J. Am. Chem. Soc..

[cit38] Fang Y., Murase T., Sato S., Fujita M. (2013). J. Am. Chem. Soc..

[cit39] Palmer L., Rebek J. (2004). Org. Biomol. Chem..

[cit40] London F. (1937). Trans. Faraday Soc..

[cit41] Grimme S. (2012). Chem.–Eur. J..

[cit42] Grimme S., Huenerbein R., Ehrlich S. (2011). ChemPhysChem.

[cit43] Xie Y., Yang H., Wang Z. U., Liu Y., Zhou H.-C., Li J.-R. (2014). Chem. Commun..

[cit44] Löffler S., Lübben J., Krause L., Stalke D., Dittrich B., Clever G. H. (2015). J. Am. Chem. Soc..

[cit45] Clever G. H., Tashiro S., Shionoya M. (2009). Angew. Chem., Int. Ed..

[cit46] Clever G. H., Kawamura W., Shionoya M. (2011). Inorg. Chem..

[cit47] Takeda T., Sugihara H., Suzuki Y., Kawamata J., Akutagawa T. (2014). J. Org. Chem..

[cit48] Saha M. L. M., Pramanik S. S., Schmittel M. M. (2012). Chem. Commun..

[cit49] Clever G. H., Shionoya M. (2010). Chem.–Eur. J..

[cit50] As opposed to our previously reported study concerning the encapsulation of the same guest inside a *D*_4h_-symmetric [Pd_2_L_4_] coordination cage, see ref. 45

[cit51] Since the addition of more than one equivalent of guest to the cage also resulted in the concomitant onset of precipitation, we were not able to determine reliable association constants for these host–guest systems

[cit52] Schulte T. R., Krick M., Asche C. I., Freye S., Clever G. H. (2014). RSC Adv..

[cit53] Becke A. D. (1993). J. Chem. Phys..

[cit54] Spackman M. A., Jayatilaka D. (2009). CrystEngComm.

[cit55] (e) Werner H.-J. , Knowles P. J., Knizia G., Manby F. R. and Schütz M., *Molpro*, version 2012.1, a package of *ab initio* programs, 2012, http://www.molpro.net

[cit56] Yoshizawa M., Klosterman J. K., Fujita M. (2009). Angew. Chem., Int. Ed..

