# Supplementary material for: Internal dynamics and guest binding of a sterically overcrowded host
Source: Chem Sci. 2016 Apr 4;7(7):4676–84. doi: 10.1039/c6sc00985a (PMC6013793; doi:10.1039/c6sc00985a)
Supplement: SC-007-C6SC00985A-s001 [file SC-007-C6SC00985A-s001.pdf]

Electronic Supplementary Material (ESI) for Chemical Science.
This journal is © The Royal Society of Chemistry 2016

Internal Dynamics and Guest Binding of a Sterically Overcrowded Host

Susanne Löffler, Jens Lübben, Axel Wuttke, Ricardo A. Mata, Michael John, Birger Dittrich, and Guido H. Clever

Contents:

1 Experimental Section **2**

1.1 Ligand Synthesis *2*

1.2 Formation of Cage [$Pd_2L^2{}_4$] *4*

1.3 Formation of Cage [$Pt_2L^2{}_4$] *4*

1.4 Synthesis of Guest Molecules *5*

2 Titration of cage [Pd_2L_4] with (bis)-anionic guests **7**

2.1 Guest molecules *7*

2.2 General procedure *7*

2.3 Titration of [$Pd_2L^2{}_4$] with bis-anionic guests *8*

2.4 Titration of cage [$Pd_2L^3{}_4$] and G^1 *15*

3 2D NMR Experiments **16**

3.1 NOESY measurements of ligand L^2 at different temperatures *16*

3.2 NOESY measurements of [$Pd_2L^2{}_4$] *17*

3.3 NOESY measurements of [$Pt_2L^2{}_4$] *19*

3.4 NOESY measurements of [G^1@$Pd_2L^2{}_4$] *20*

3.5 NOESY measurements of [G^2@$Pd_2L^2{}_4$] *21*

3.6 EXSY Measurements *23*

3.7 EXSY measurement of a 1:1 mixture of [$Pd_2L^2{}_4$] and [G^2@$Pd_2L^2{}_4$] *24*

3.8 Addition of pyridine to [$Pd_2L^2{}_4$] *26*

4 Cyclic Voltammetry **27**

5 X-Ray Data **27**

6 Hirshfeld surface analysis **29**

7 Computational details **30**

7.1 Ligand flipping *30*

7.2 Guest binding *30*

7.3 Dispersion Interaction Density plots *31*

8 References **32**

1 Experimental Section

1.1 Ligand Synthesis[1]

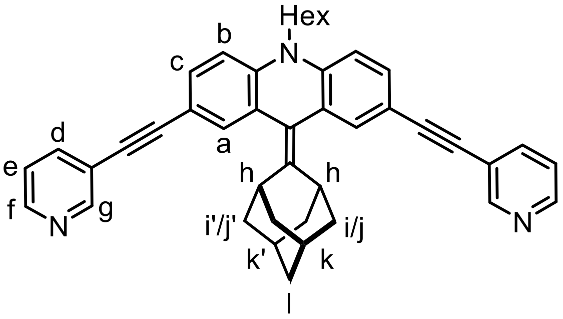

Under a nitrogen atmosphere, titanium(III)-chloride (286.0 mg, 2.66 mmol, 17 eq.) in THF (8 mL) was cooled to 0 °C, lithium aluminium hydride (50.4 mg, 1.33 mmol, 9 eq.) was added and stirred for 10 min at 0 °C. Triethylamine (135.5 mg, 1.33 µmol, 9 eq.) was added dropwise at room temperature and the mixture was stirred for 1 h at 90 °C. 10-hexyl-2,7-bis(pyridine-3-ylethinyl)lacrid-9(10*H*)-one [2] (ligand **L**[1]) (74.4 mg, 155 µmol, 1.0 eq.) and 2-adamantone (23.2 mg, 155 µmol, 1.0 eq.) in THF (5 mL) were added dropwise and the mixture was stirred at 90 °C. After 18 h, the mixture was cooled to room temperature and filtered over Celite®. The solvent was removed *in vacuo* and the residue was purified using flash column chromatography on silica gel (0–2.5% MeOH in $CHCl_3$) to give the product as a yellow solid (79.4 mg, 132 µmol, 85%).

^{1}H NMR (500 MHz, 273 K, CD_3CN): δ (ppm) = 8.72 (dd, ^{4}J = 2.1 Hz, ^{4}J = 0.9 Hz, 2H), 8.53 (dd, ^{3}J = 4.9 Hz, ^{4}J = 1.7 Hz, 2H), 7.86 (ddd, ^{3}J = 7.9 Hz, ^{4}J = 2.2 Hz, ^{4}J = 1.7 Hz, 2H), 7.42 (dd, ^{3}J = 8.5 Hz, ^{4}J = 2.0 Hz, 2H), 7.36 (ddd, ^{3}J = 7.9 Hz, ^{4}J = 4.9 Hz, ^{5}J = 0.8 Hz), 7.34 (d, ^{3}J = 1.9 Hz, 2H), 7.17 (d, ^{3}J = 8.6 Hz, 2H), 4.04 (t, ^{3}J = 6.9 Hz, 2H, NCH_2), 3.32 (s, 2H), 2.10–2.02 (m, 5H), 1.90 (s, 2H), 1.85–1.81 (m, 1H) 1.80–1.75 (m, 4H, hexyl-CH_2, adamantyl-*H*), 1.50–1.42 (m, 2H), 1.38–1.30 (m, 2H, hexyl-CH_2), 1.29–1.22 (m, 4H, hexyl-CH_2), 0.84 (t, ^{3}J = 6.9 Hz, 3H, hexyl-CH_3).

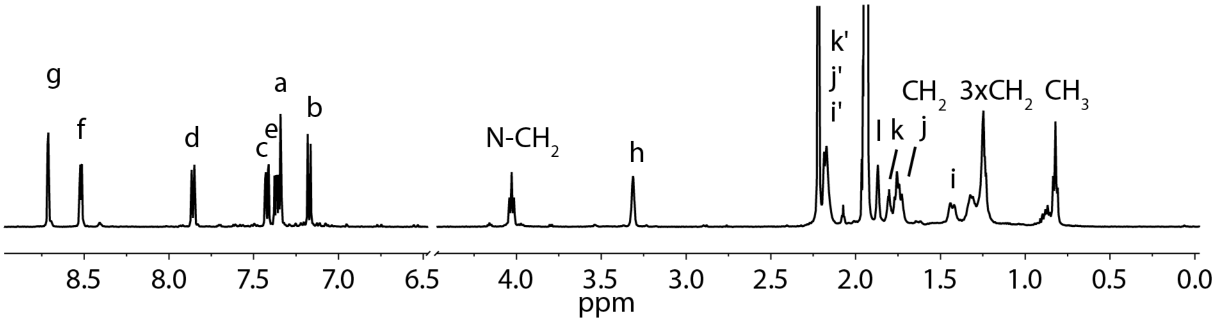

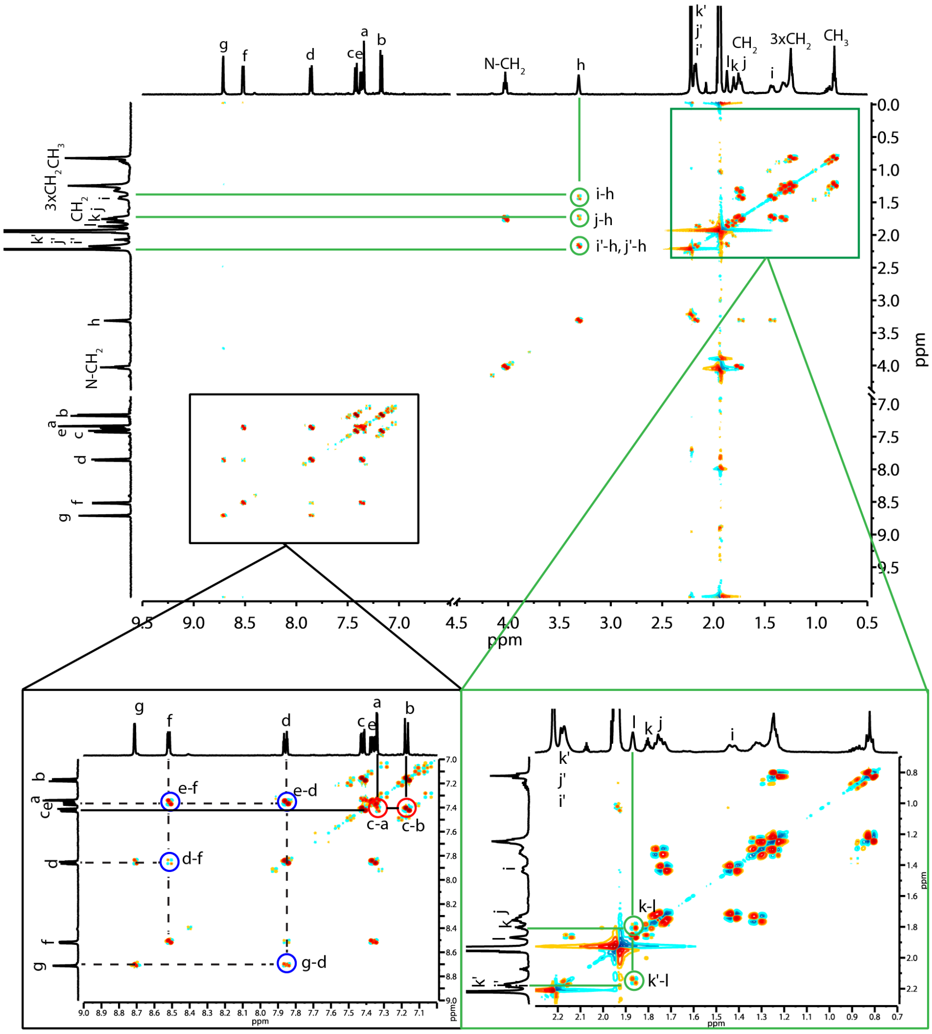

^{1}H-^{1}H COSY spectrum of ligand **L²** (500 MHz, CD_3CN, 273 K). Cross Peaks are highlighted: <u>Blue:</u> pyridine hydrogen, <u>red:</u> acridone protons, <u>green</u>: adamantyl hydrogen atoms.

^{13}C NMR (75 MHz, 273 K, CD_3CN): δ (ppm) = 151.66, 148.51, 146.32, 143.45, 138.08, 130.32, 130.06, 125.84, 123.45, 120.41, 114.10, 113.87, 92.72, 85.17, 45.11, 39.41, 38.61, 36.37, 32.19, 31.19, 28.20, 27.49, 25.97, 22.49, 13.40.

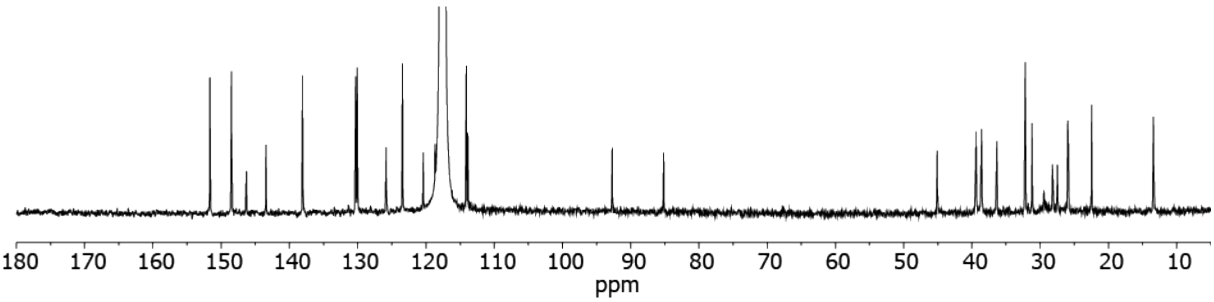

ESI HRMS ($C_{43}H_{41}N_3$): measured: 600.3375

calculated: 600.3373

IR (ATR): $\tilde{\nu}$ (cm^{-1}) = 2954, 2920, 2849, 2206, 1733, 1577, 1462, 1373, 1259, 1019, 799, 702.

Melting Point: 67.5 °C

1.2 Formation of Cage [$Pd_2L^2_4$]

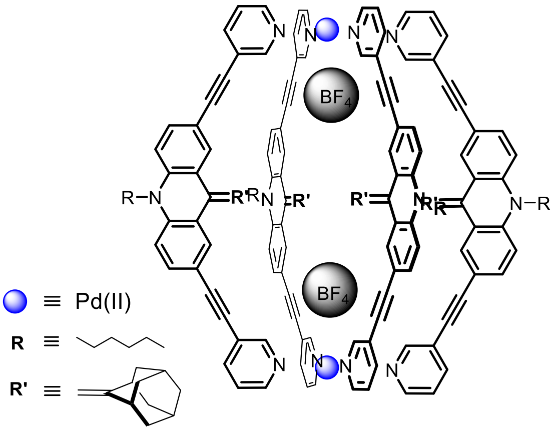

The cage compound was formed in quantitative yields by heating a mixture of the ligand **L²** (804 µL, 1.35 mg, 2.3 µmol, 1.0 eq.) in CD_3CN (804 µL) and [$Pd(CH_3CN)_4(BF_4)_2$] (1.13 µmol, 0.5 eq, 80.4 µL of a 15 mM solution in CD_3CN) at 70 °C for 15 min to give a 0.70 mM solution of the [$Pd_2L^2_4$] cage.

^{1}H NMR (500 MHz, 298 K, CD_3CN): δ (ppm) = 9.12 (s, 8H), 8.86 (d, ^3J = 5.8 Hz, 8H), 8.07 (dt, ^3J = 8.1 Hz, ^4J = 1.6 Hz, 8H), 7.58 (dd, ^3J = 8.0 Hz, ^3J = 5.7 Hz, 8H), 7.48 (dd, ^3J = 8.5 Hz, ^4J = 1.8 Hz, 8H), 7.35 (d, ^4J = 2.0 Hz, 8H), 7.19 (d, ^3J = 8.5 Hz, 8H), 4.01 (t, ^3J = 6.9 Hz, 8H, NCH_2), 3.17 (s, 8H), 2.00 (8 H), 1.75–1.66 (m, 16 H, CH_2), 1.47–1.43 (m, 8H), 1.35–1.32 (m, 8H), 1.20–1.15 (m, 40H, CH_2), 0.75 (t, ^3J = 6.9 Hz, 12H, CH_3,), 0.59 (s, 8H).

Overlapping signals in the aliphatic region could be assigned via 2D NMR spectroscopy.

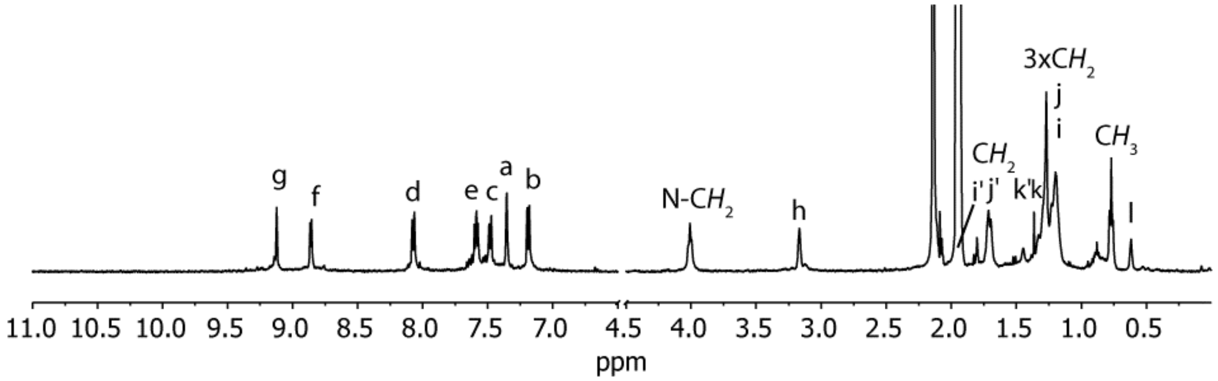

^{19}F NMR (282 MHz, 298 K, CD_3CN): δ (ppm) = −151.56 (sharp, BF_4^-).

ESI HRMS ([$C_{172}H_{164}N_{12}Pd_2(BF_4)_2$]$^{2+}$: exp.: 1392.6103

calc.: 1392.6100

IR (ATR): $\tilde{\nu}$ (cm^{-1}) = 3620, 2921, 2858, 2261, 2211, 1588, 1469, 1104, 1038, 832, 690.

1.3 Formation of Cage [$Pt_2L^2_4$]

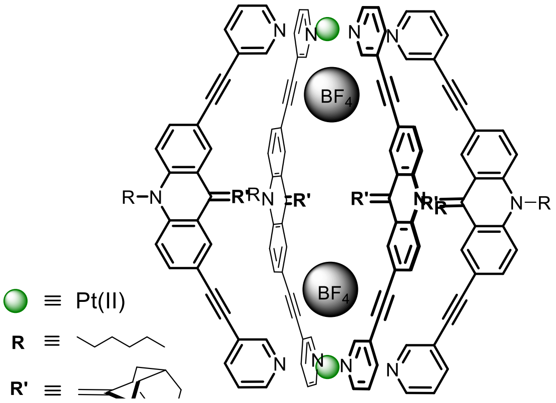

Ligand **L²** (1.68 mg, 2.8 mmol, 1.0 eq.), [$PtCl_2(CH_3CN)_2$] (0.58 mg, 1.4 mmol, 0.5 eq.) and $AgClO_4$ (0.58 mg, 2.8 mmol, 1.0 eq.) were heated to 80 °C in acetonitrile (1 mL) for 3 days and the precipitated silver chloride was removed by filtration.

The [Pt_2**L^2**$_4$] cage was formed quantitatively as a 0.70 mM solution.

^{1}H-NMR (500 MHz, 298 K, CD_3CN): δ (ppm) = 9.23 (s, 8H), 8.86 (d, ^3J = 5.8 Hz, 8H), 8.06 (dt, ^3J = 8.1 Hz, ^4J = 1.6 Hz, 8H), 7.58 (dd, ^3J = 8.0 Hz, ^3J = 5.7 Hz, 8H), 7.48 (dd, ^3J = 8.5 Hz, ^4J = 1.8 Hz, 8H), 7.39 (d, ^4J = 2.0 Hz, 8H), 7.18 (d, ^3J = 8.5 Hz, 8H), 4.01 (t, ^3J = 6.9 Hz, 8H, NCH_2), 3.17 (s, 8H), 2.04 (8 H), 1.75–1.66 (m, 16 H, CH_2), 1.59–1.52 (m, 8H), 1.35–1.15 (m, 44H, CH_2), 0.77 (t, ^3J = 6.9 Hz, 12H, CH_3), 0.49 (s, 8H).

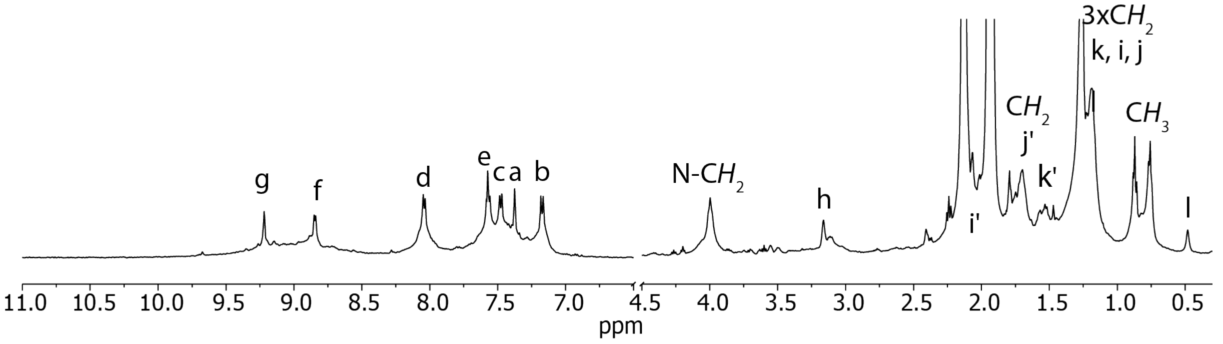

ESI-HRMS ([$C_{172}H_{164}N_{12}Pt_2(ClO4)_2]^{2+}$: measured: 1494.0765

calculated: 1494.0746

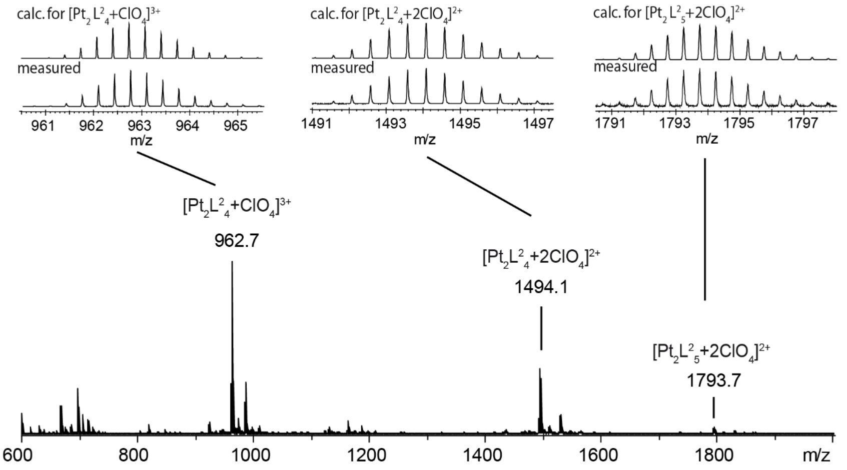

IR (ATR): $\tilde{\nu}$ (cm^{-1}) = 3620, 2921, 2858, 2261, 2211, 1588, 1469, 1104, 1038, 832, 690.

1.4 Synthesis of Guest Molecules

The tetrabutylammonium salts of the guests were obtained according to a previously reported procedure.[3] All aromatic starting materials were obtained commercially as sodium salts or free acids in the highest available purity. The ferrocene 1,1'-disulfonic acid was prepared following the procedure reported by *Jones* and coworkers.[4]

The sodium salts (1.0 eq.) were dissolved in methanol and an acidic cation exchange resin (DOWEX®50WX8) was added and stirred for 1–2 h, filtered and the solvent evaporated to obtain the free acids. To a solution of the disulfonic acids in methanol, tetrabutylammonium hydroxide (10 % in MeOH, 2.0 eq.) was added and the pH was adjusted to 7. After

evaporation of the solvent, the crude residue was mixed with $CHCl_3$ and then filtered to remove any remaining solids. Finally, the solvent was removed *in vacuo* to yield the tetrabutylammonium salts. The purity of the compounds and the correct cation : anion ratio was verified by ^{1}H NMR spectroscopy.

The preparation of the potassium@18-crown-6 salts was carried out as follows: To a solution of the bis-sulfonic acid (1.0 eq.) in methanol, a solution of potassium hydroxide (1 M in MeOH, 2.0 eq.) was added until the pH was adjusted to 7. Then, a stoichiometric solution of 18-crown-6 (1 M in MeOH, 2.0 eq.) was added and the solvent was removed *in vacuo.* The potassium 18-crown-6 salts were obtained in good yields (83−95 %) after recrystallization from methanol.

2 Titration of cage [Pd_2**L_4**] with (bis)-anionic guests

2.1 Guest molecules

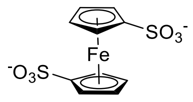

G^1

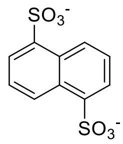

G^2

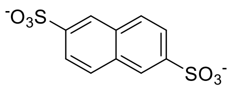

G^3

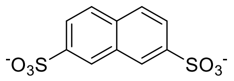

G^4

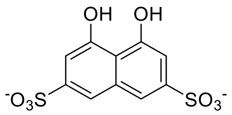

G^5

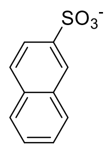

G^6

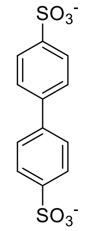

G^7

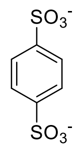

G^8

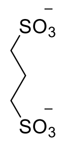

G^9

2.2 General procedure

The host-guest complexes were formed by titrating a solution of the bis-sulfonate guests (as their tetrabutylammonium or potassium@18-crown-6 salts in CD_3CN, 17.5 mM) into a solution of the cage [Pd_2**L_4**] (0.70 mM, 500 µL, CD_3CN) in an NMR tube. The NMR spectra were recorded immediately after briefly shaking the solution.

2.3 Titration of $[Pd_2L^2_4]$ with bis-anionic guests

2.3.1 Addition of G^1

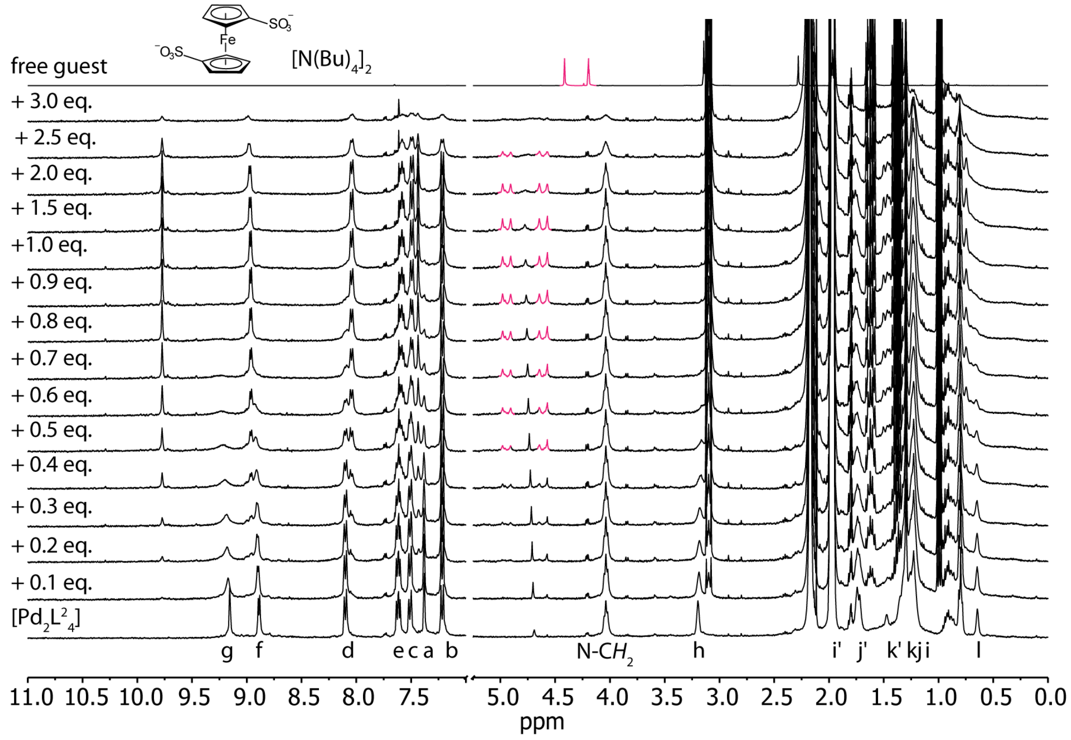

Figure SI 1 ^{1}H NMR titration (400 MHz, 298 K, CD_3CN) of $[Pd_2\mathbf{L}^2_4]$ with $(NBu_4)_2\mathbf{G}^1$. Upon addition of one equivalent of guest $\mathbf{G}^1$ the $[Pd_2\mathbf{L}^2_4]$ cage transforms into $[\mathbf{G}^1@Pd_2\mathbf{L}^2_4]$. Excess addition of $\mathbf{G}^1$ leads to disaggregation of the cage.

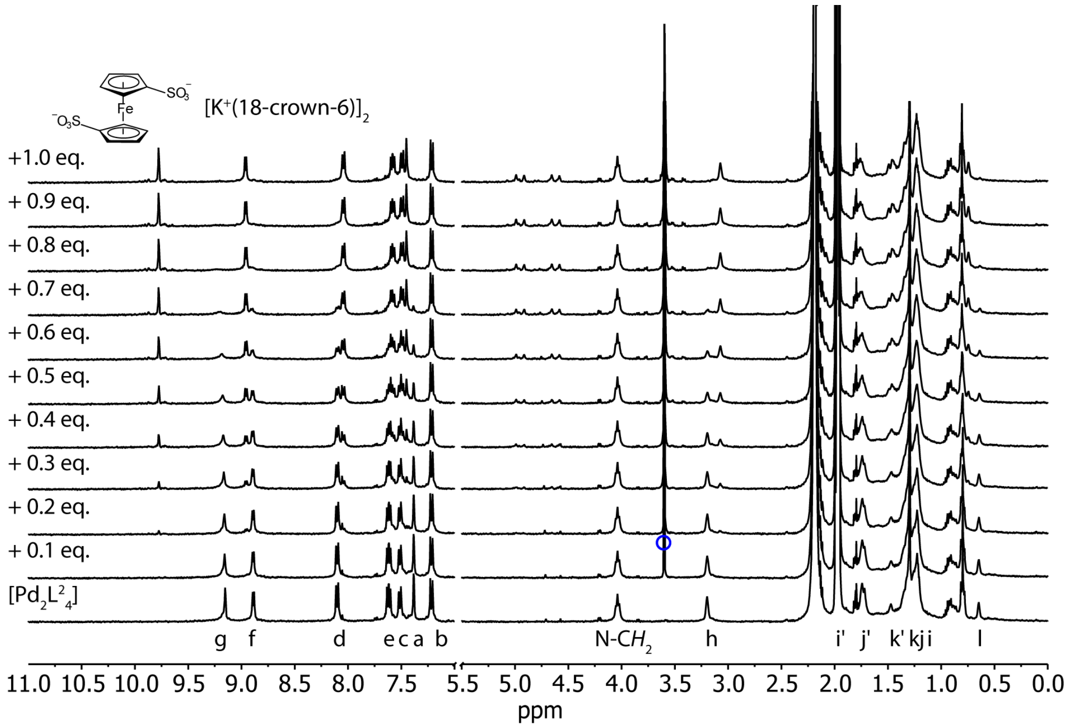

Figure SI 2 ^{1}H NMR titration (400 MHz, 298 K, CD_3CN) of $[Pd_2\mathbf{L}^2_4]$ with $[K^+@(18\text{-crown-6})]_2\mathbf{G}^1$. Upon addition of one equivalent of guest $\mathbf{G}^1$ the $[Pd_2\mathbf{L}^2_4]$ cage transforms into $[\mathbf{G}^1@Pd_2\mathbf{L}^2_4]$. The blue circle indicates the crow ether signal.

2.3.2 Addition of G^2

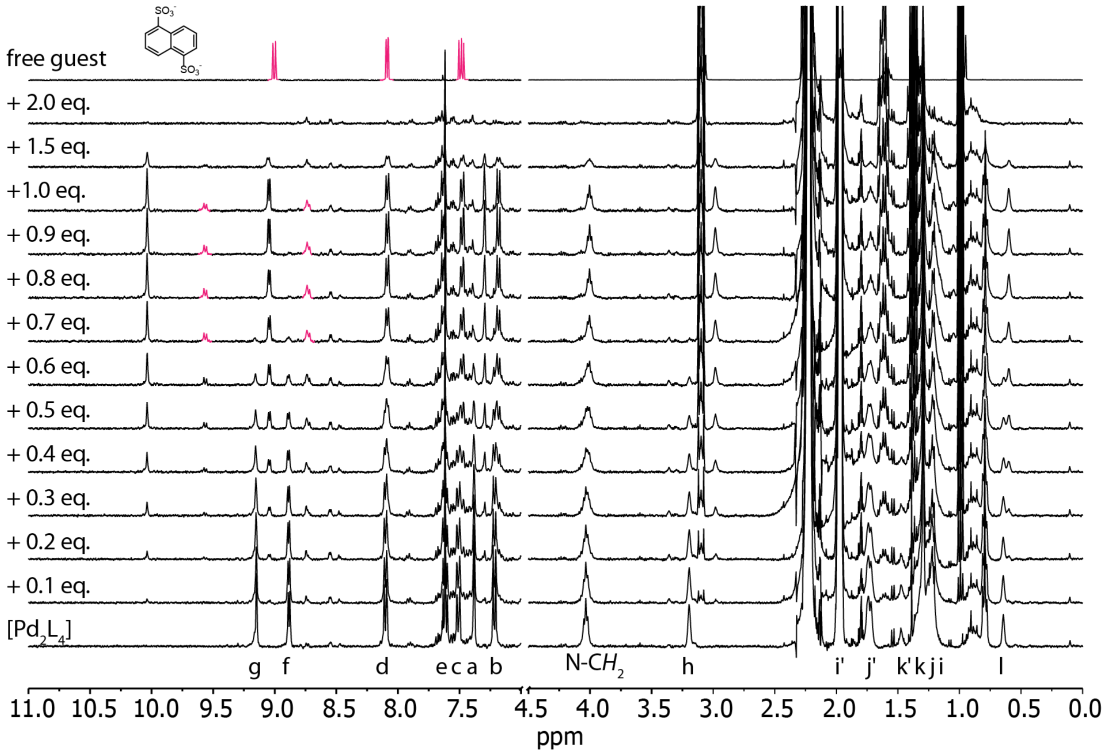

Figure SI 3 ^1H NMR titration (400 MHz, 298 K, CD_3CN) of [Pd_2**L**$^2{}_4$] with $(NBu_4)_2$**G**^{2}. Upon addition of 1.0 eq. of guest **G**^{2} the [Pd_2**L**$^2{}_4$] cage transforms into the host-guest complex [**G**^{2}@Pd_2**L**$^2{}_4$]. Excess addition of **G**^{2} leads to precipitation and disassembly of the cage. Signals of encapsulated guest **G**^{2} are highlighted in magenta.

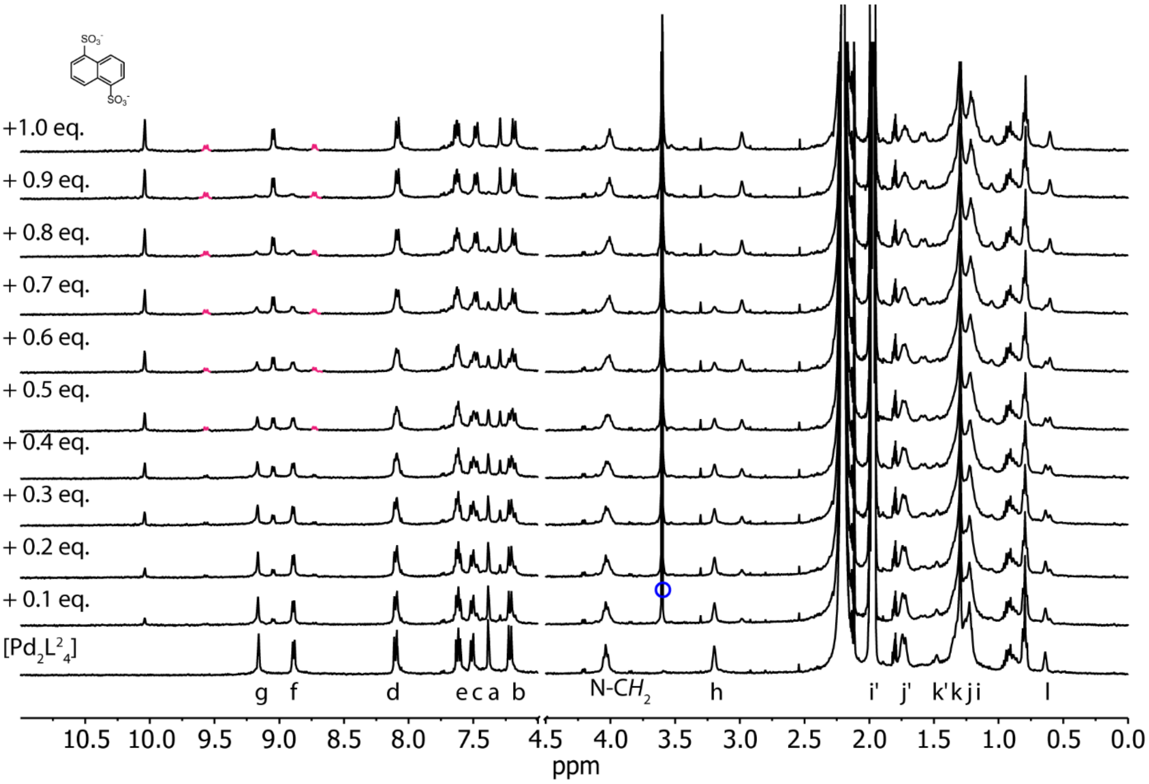

Figure SI 4 ^1H NMR titration (400 MHz, 298 K, CD_3CN) of [Pd_2**L**$^2{}_4$] with [K^+@(18-crown-6)]$_2$**G**^{2}. Upon addition of 1.0 eq. of guest **G**^{2} to the [Pd_2**L**$^2{}_4$] cage the new host guest complex [**G**^{2}@Pd_2**L**$^2{}_4$] is formed. The blue circle indicates the crow ether signal.

2.3.3 Addition of G^3

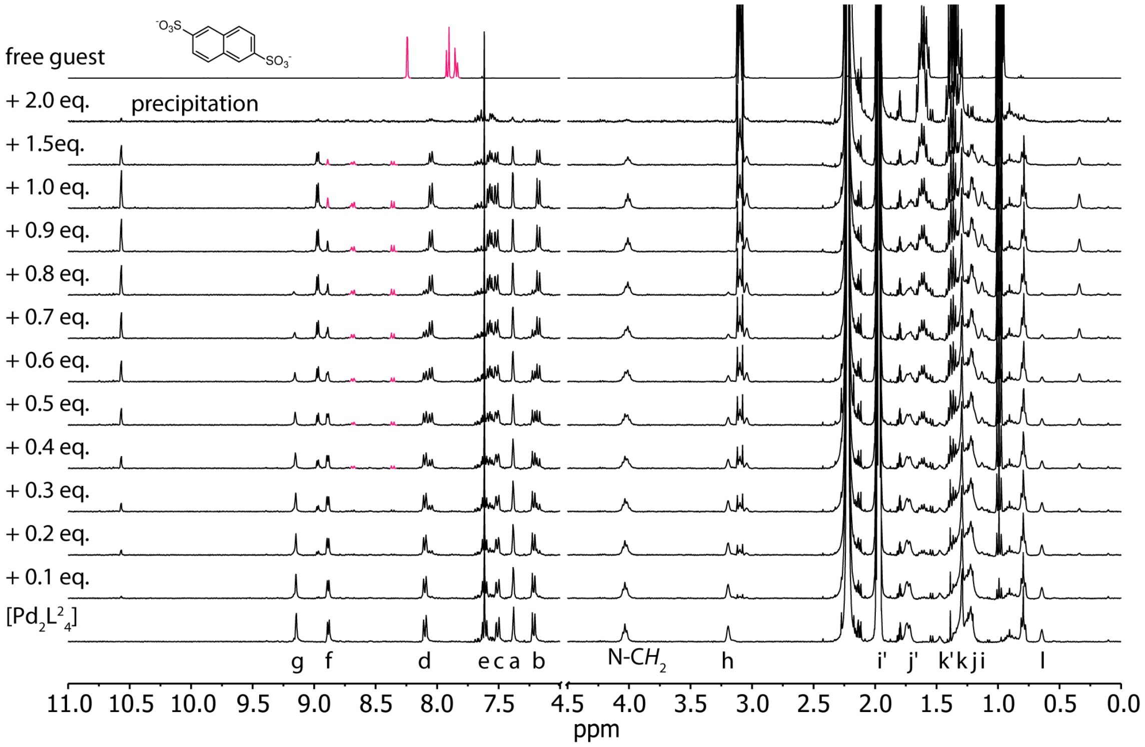

Figure SI 5 ^{1}H NMR titration (400 MHz, 298 K, CD_3CN) of [Pd_2**L**$^{\mathbf{2}}_4$] with $(NBu_4)_2$**G**$^{\mathbf{3}}$. Excess addition of guest molecules leads to decomposition of the host guest complex.

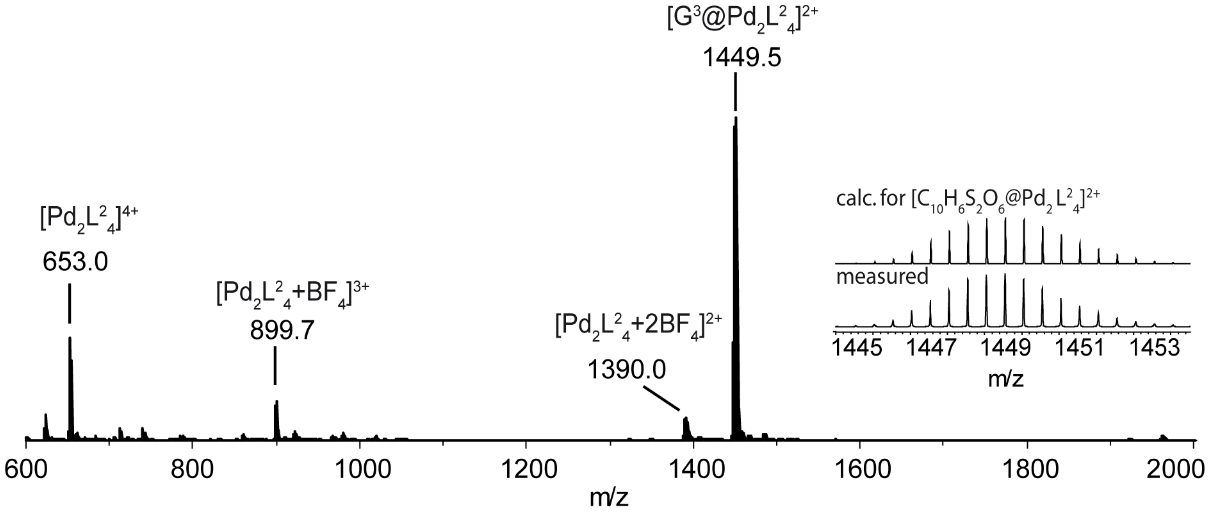

Figure SI 6 ESI-MS of [**G**$^{\mathbf{3}}$@Pd_2**L**$^{\mathbf{2}}_4$].

2.3.4 Addition of G^4

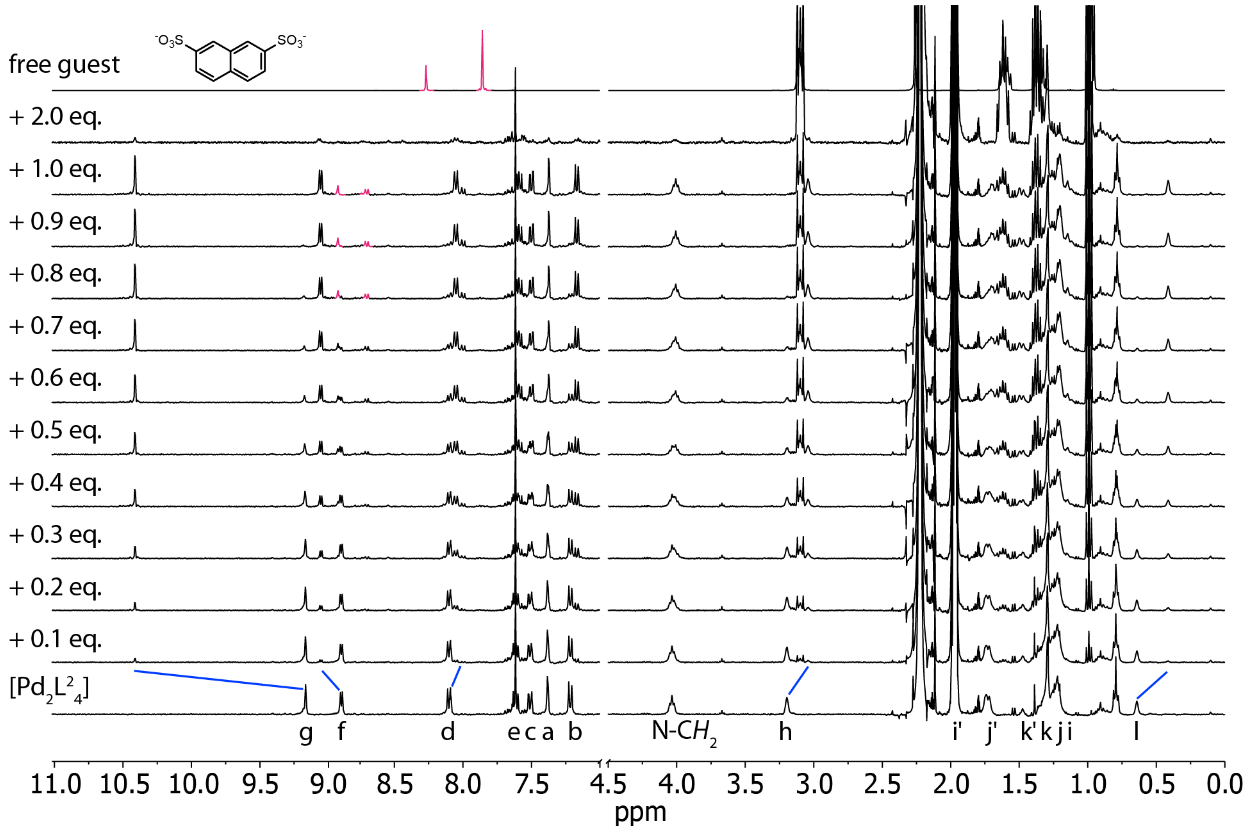

Figure SI 7 ^{1}H NMR titration (400 MHz, 298 K, CD_3CN) of [Pd_2**L**$^2{}_4$] with $(NBu_4)_2$**G**^{4}. Upon addition of 1.0 eq. of guest **G**^{4} to the [Pd_2**L**$^2{}_4$] cage the new species [**G**^{4}@Pd_2**L**$^2{}_4$] is formed.

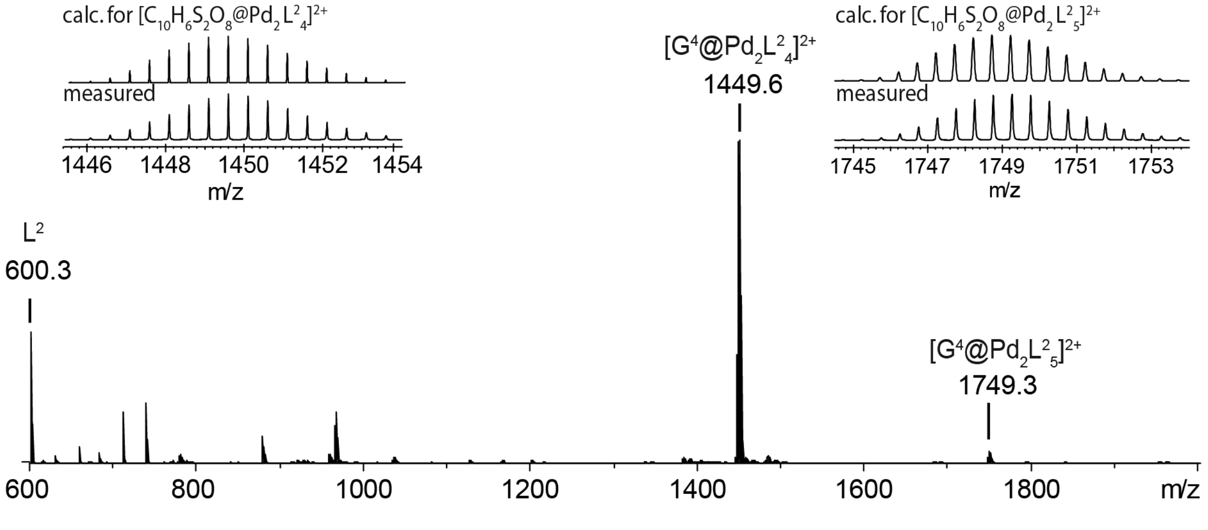

Figure SI 8 ESI-MS of [**G**^{4}@Pd_2**L**$^2{}_4$].

2.3.5 Addition of G^5

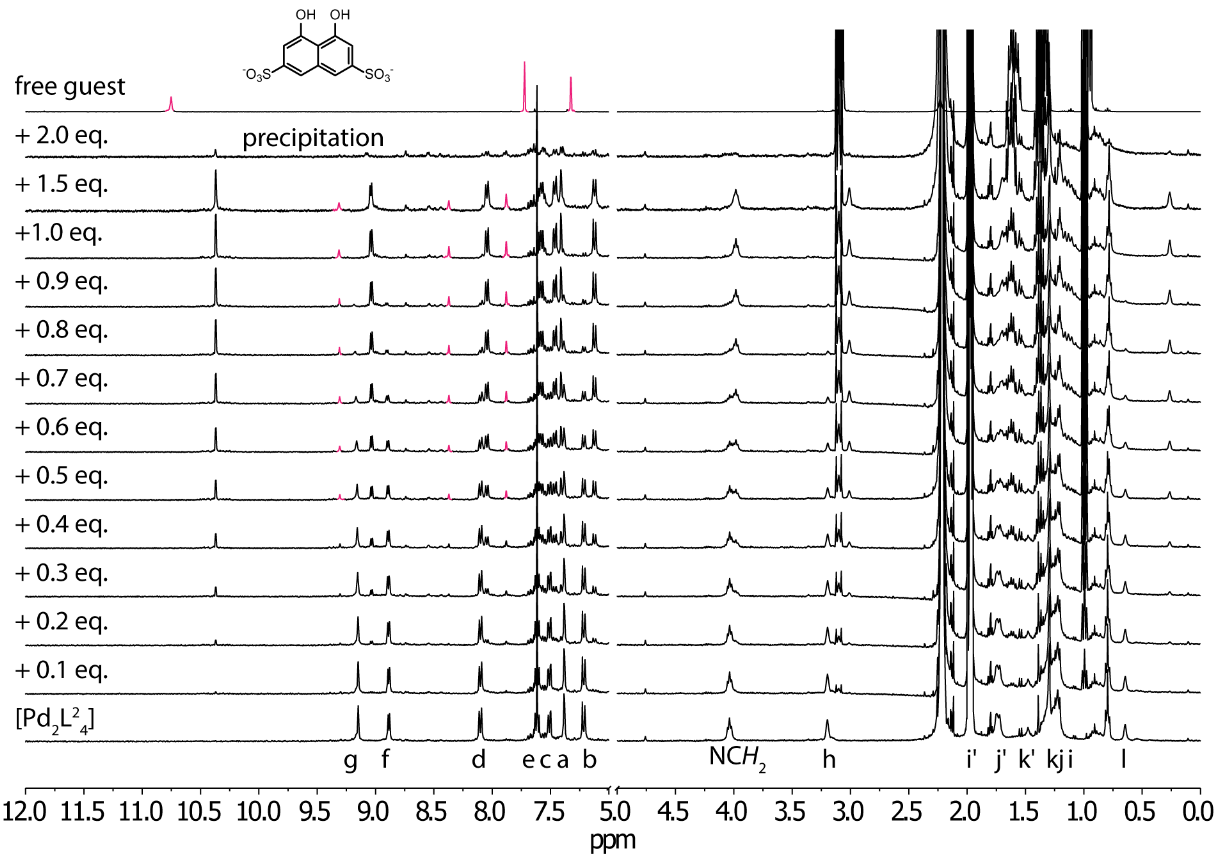

Figure SI 9 ^{1}H NMR titration (400 MHz, 298 K, CD_3CN) of [$Pd_2\mathbf{L}^2_4$] with $(NBu_4)_2\mathbf{G}^5$. Upon addition of one equivalent of guest $\mathbf{G}^5$ to the [$Pd_2\mathbf{L}^2_4$] cage the new species [$\mathbf{G}^5$@$Pd_2\mathbf{L}^2_4$] is formed. Excess addition of $\mathbf{G}^5$ leads to complete disaggregation of the cage and precipitation was observed in the NMR tube.

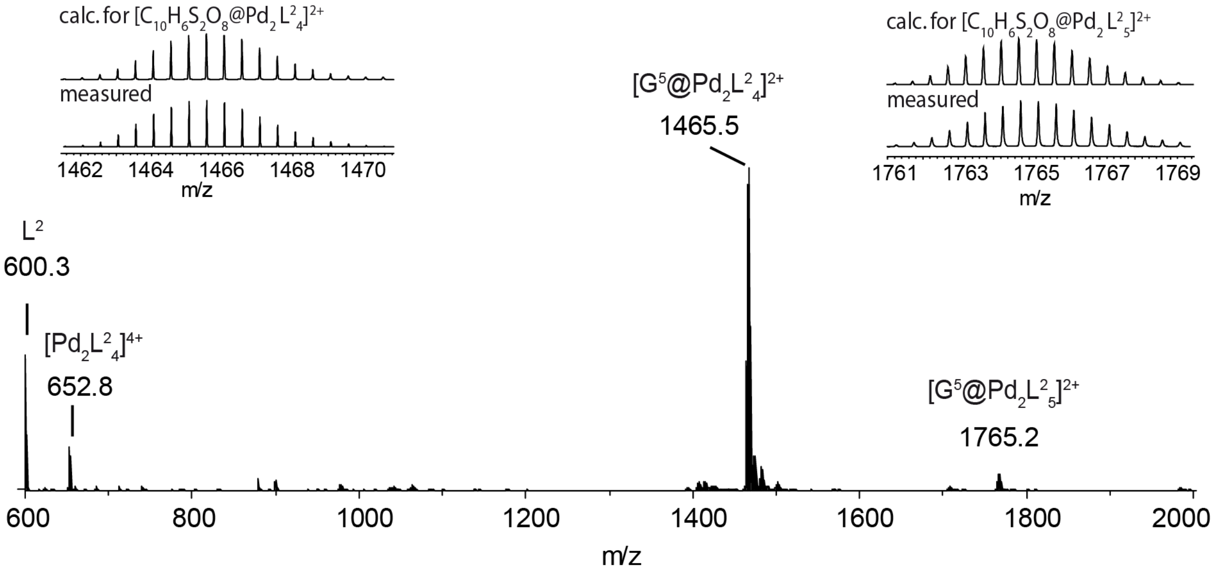

Figure SI 10 ESI-MS of [$\mathbf{G}^5$@$Pd_2\mathbf{L}^2_4$].

2.3.6 Addition of G^6

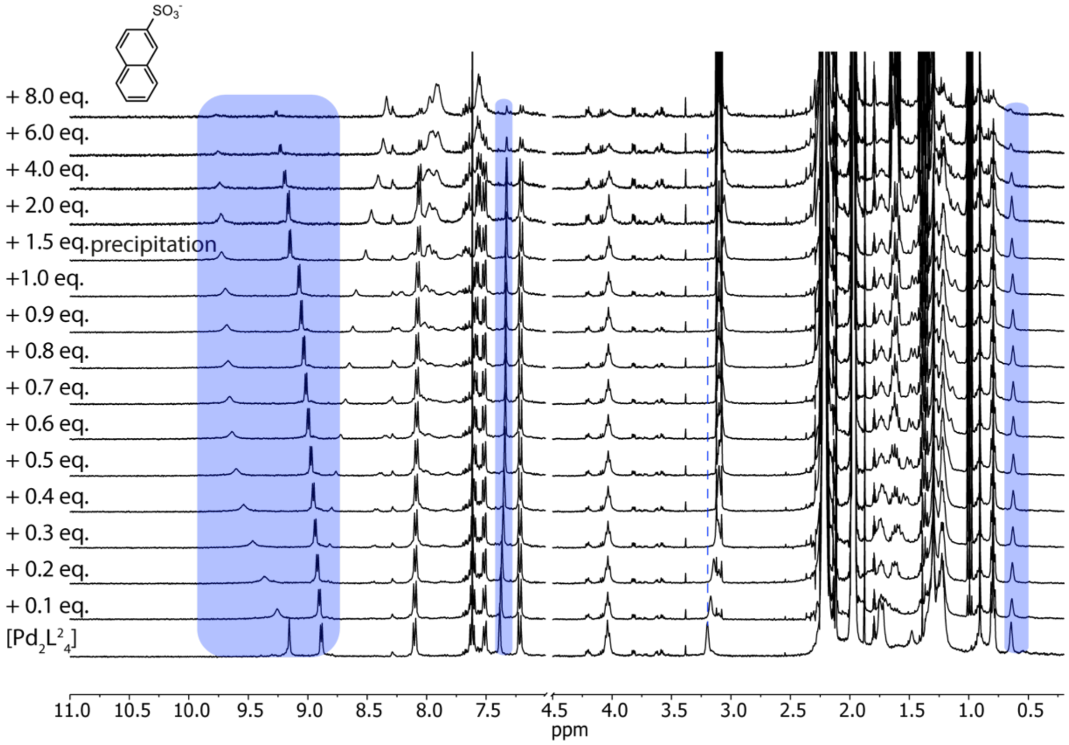

Figure SI 11 ^{1}H NMR titration (400 MHz, 298 K, CD_3CN) of [Pd_2**L**$^{\mathbf{2}}{}_4$] with $(NBu_4)_2$**G**$^{\mathbf{7}}$.

2.3.7 Addition of G^7

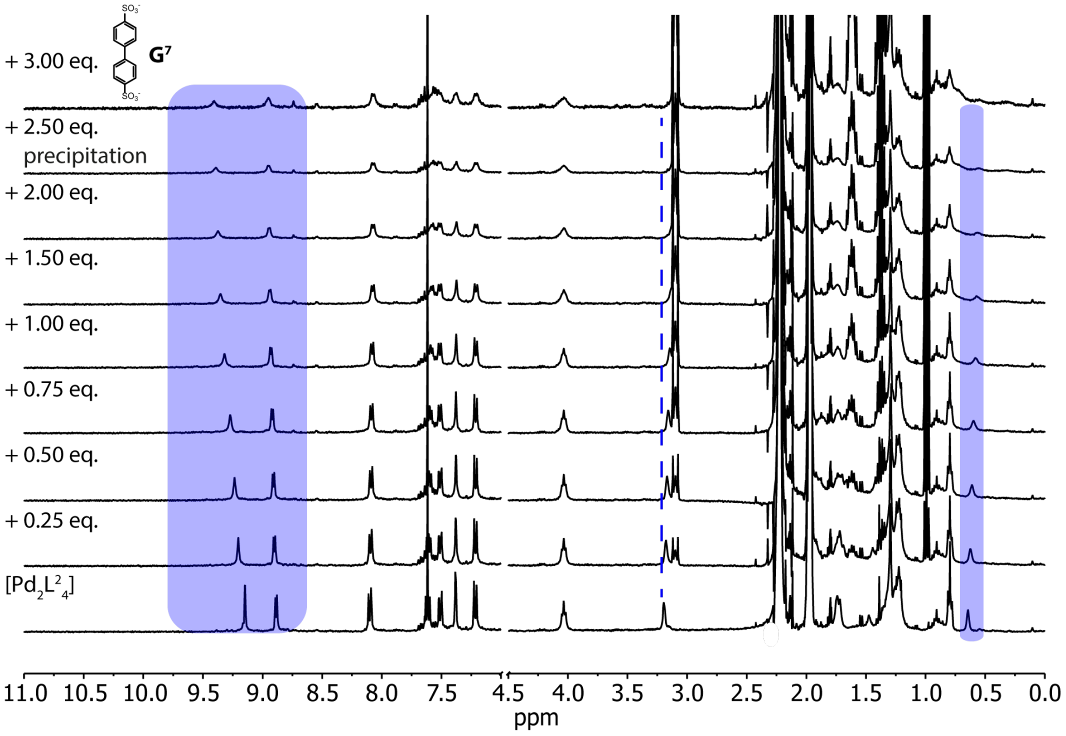

Figure SI 12 ^{1}H NMR titration (400 MHz, 298 K, CD_3CN) of [Pd_2**L**$^{\mathbf{2}}{}_4$] with $(NBu_4)_2$**G**$^{\mathbf{7}}$. The chemical shifts of the inward pointing protons changed gradually, due to fast exchange of the guest.

2.3.8 Addition of G[8]

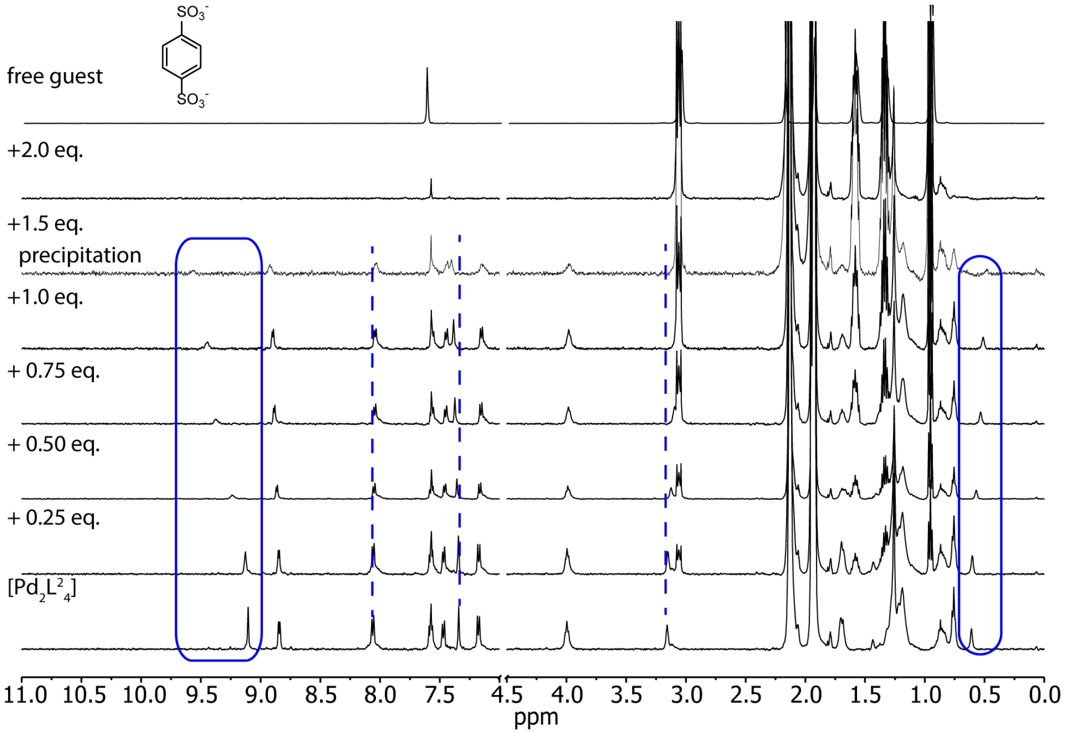

Figure SI 13 ^{1}H NMR titration (400 MHz, 298 K, CD_3CN) of [Pd_2**L**$^{2}_{4}$] with $(NBu_4)_2$**G**^{8}. The chemical shifts of the inward pointing protons changed gradually, due to fast exchange of the guest.

2.3.9 Addition of G[9]

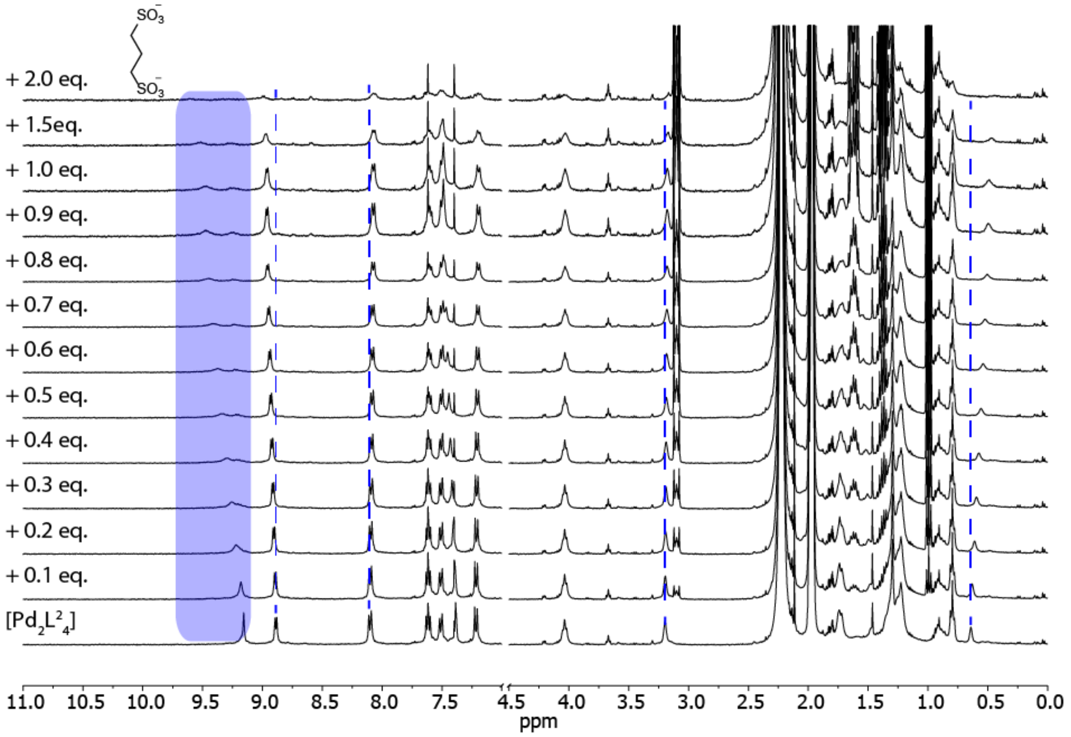

Figure SI 14 ^{1}H NMR titration (400 MHz, 298 K, CD_3CN) of [Pd_2**L**$^{2}_{4}$] with $(NBu_4)_2$**G**^{9}. The chemical shifts of the inward pointing protons changed gradually, due to fast exchange of the guest.

2.4 Titration of cage [$Pd_2L^3_4$] and G^1

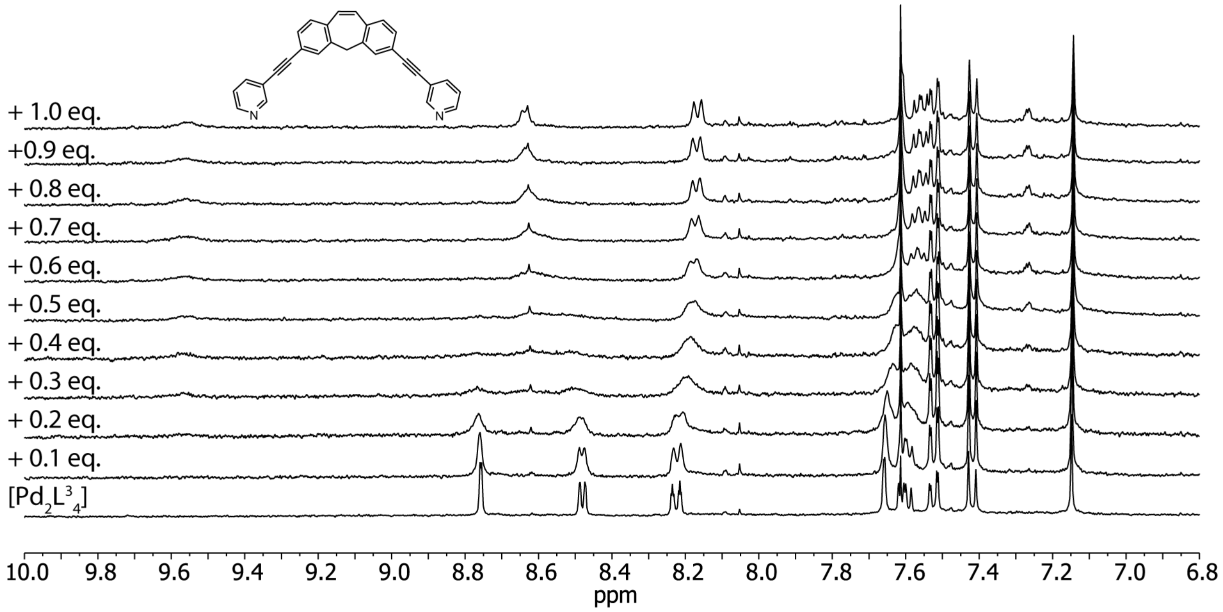

Figure SI 15 ^{1}H NMR titration (400 MHz, 298 K, CD_3CN) of [Pd_2**L^3**$_4$] with [K^+-(18-crown-6)]$_2$**G^1**. Gradual shifting of the signals indicates fast exchange. The exchange rate of a 1:1 sample of [Pd_2**L^3**$_4$] and [G^1@Pd_2**L^3**$_4$] could be given through line shape fitting to k ~ 90s $^{-1}$.

3 2D NMR Experiments

3.1 NOESY measurements of ligand L^2 at different temperatures

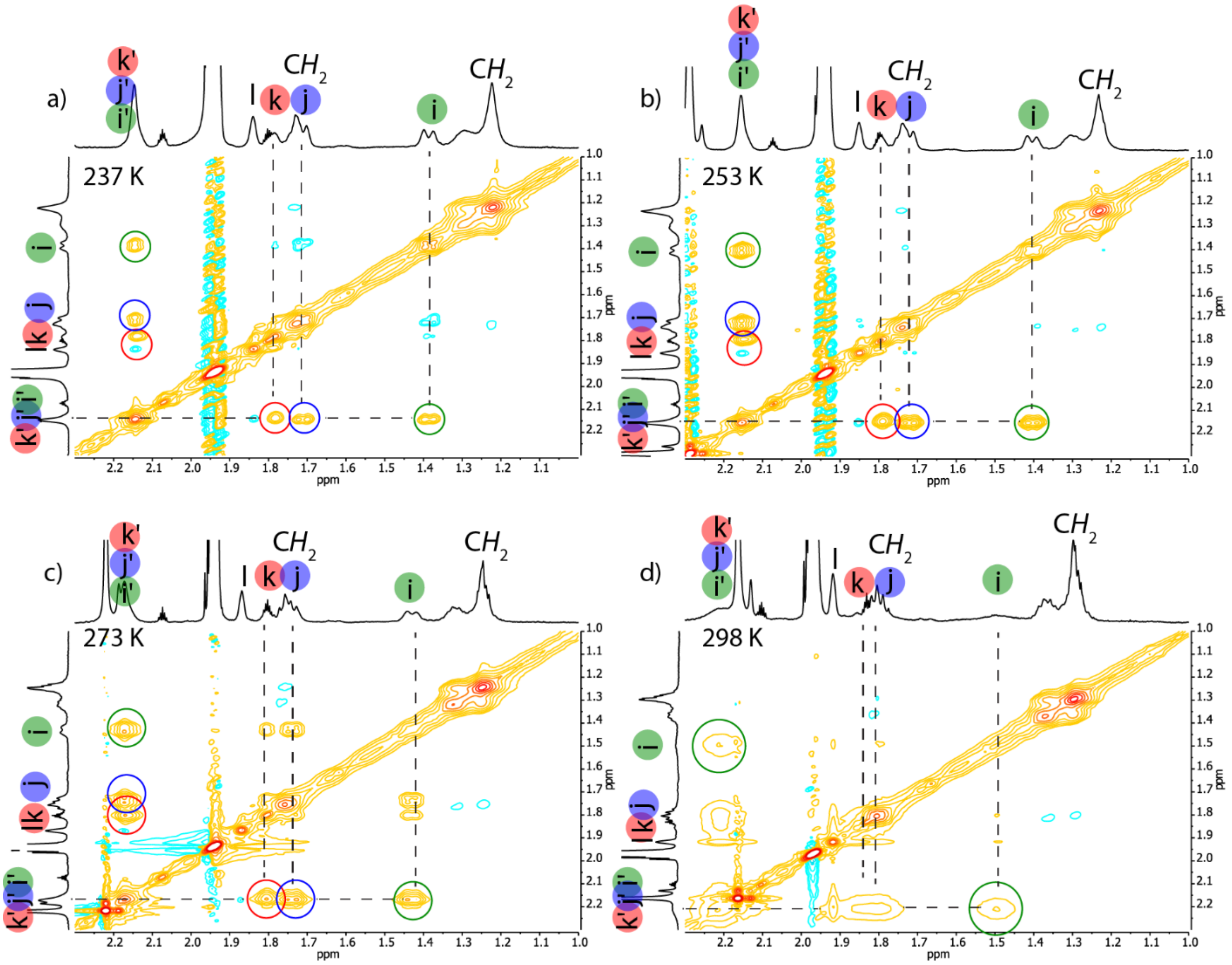

Figure SI 16 ^1H-^1H NOESY spectrum of ligand $\mathbf{L^2}$ (500 MHz, CD_3CN) at different temperatures: a) 237 K, b) 253 K, c) 273 K and d) 298 K. The cross-peaks indicate the exchange between the inward (i', j' and k') and outward (i, j, k) pointing hydrogen atoms and are increasing with rising temperature. This indicates the adamantly group is undergoing a rapid flipping motion.

Integration of the signals yielded the exchange or “flipping rate” of the adamantly group within the ligand at different temperatures (Eyring plot analysis see below):

T (K)	***k*** (s^{-1})
237	0.18
253	0.90
273	14
298	150

3.2 NOESY measurements of $[Pd_2L^2_4]$

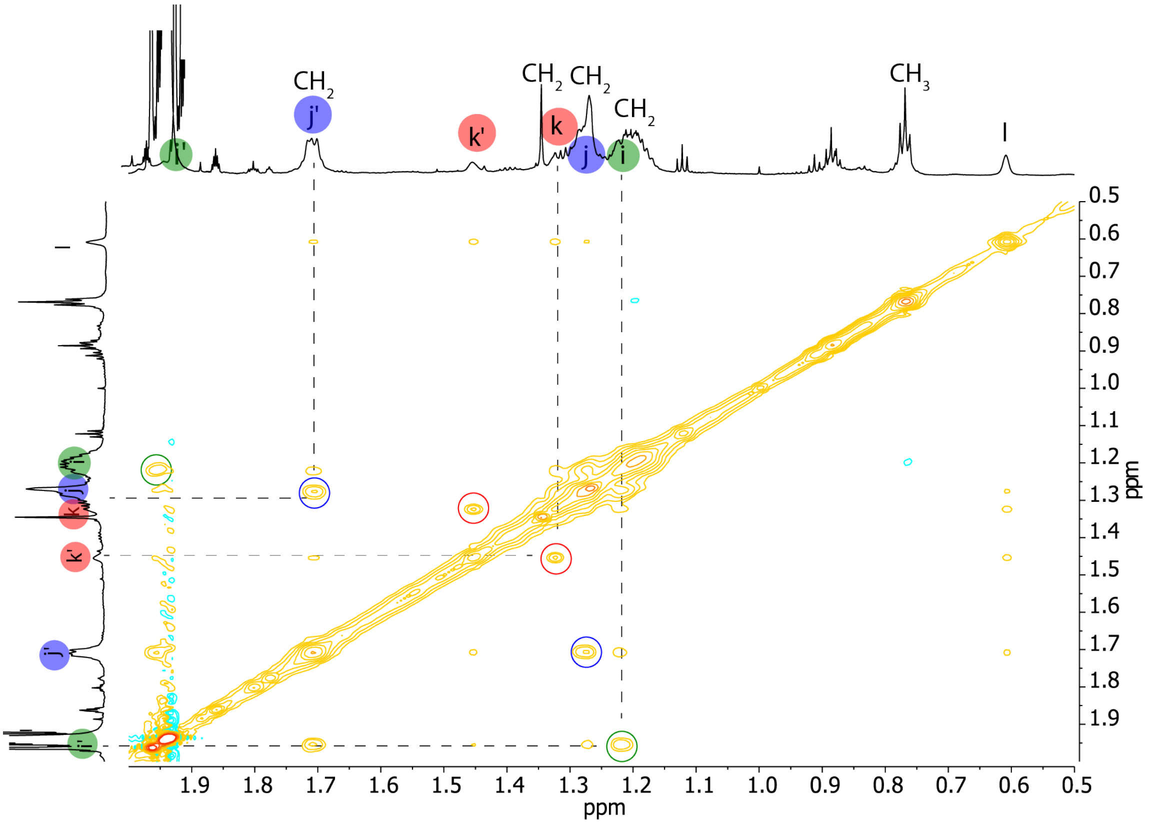

Figure SI 17 ^{1}H-^{1}H NOESY spectrum of $[Pd_2\mathbf{L}^2_4]$ (900 MHz, 298 K, mixing time 0.5 s, CD_3CN). Exchange signals between related protons are highlighted as followed: i/i' (green), j/j' (blue), k/k' (red). Integration of the inner and outer adamantyl signals determined the exchange rate as: 0.32 s^{-1}.

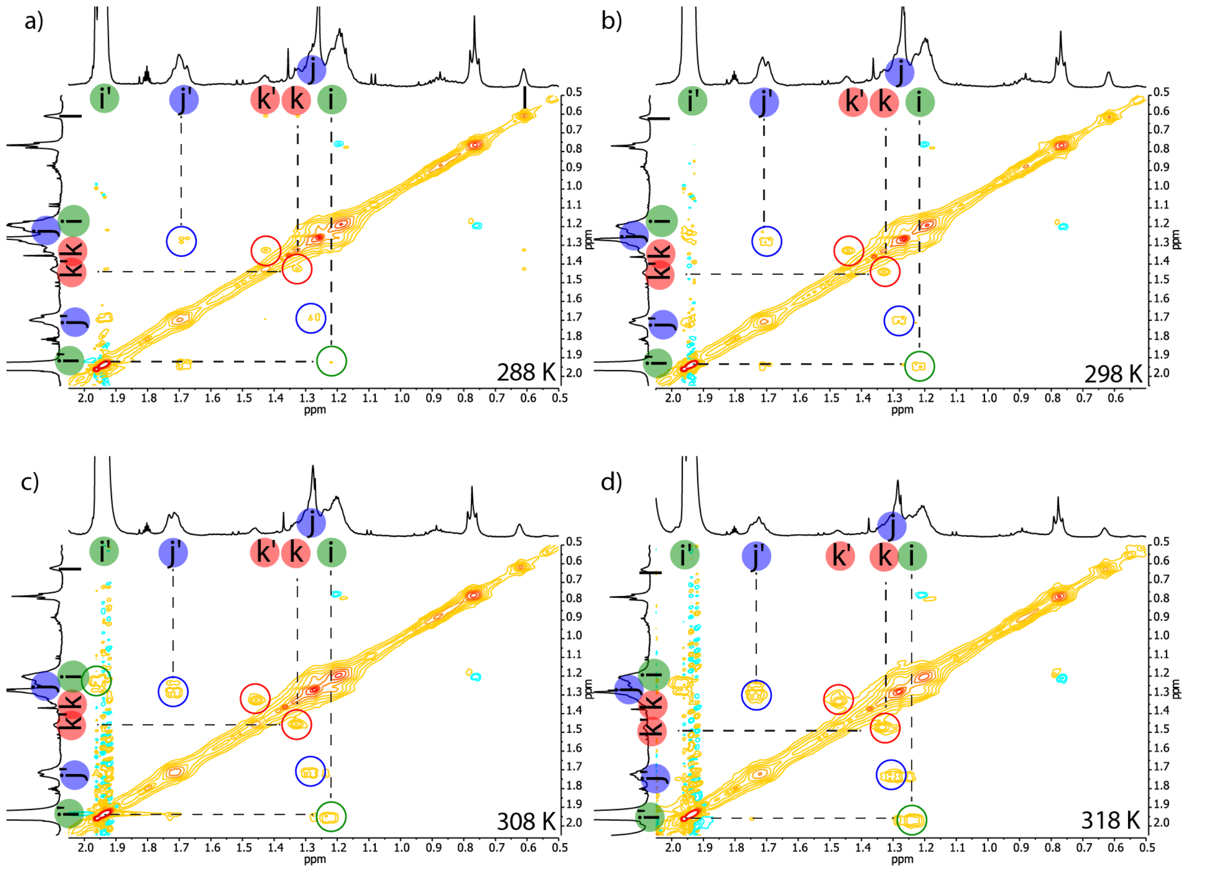

Figure SI 18 ^{1}H-^{1}H NOESY spectra of cage $[Pd_2\mathbf{L}^2_4]$ (500 MHz, CD_3CN, 0.5 s mixing time) at different temperatures: a) 288 K, b) 298 K, c) 308 K and d) 318 K. The intensities of the highlighted exchange signals between inward (i', j' and k') and outward (i, j, k) pointing protons are increasing with rising temperature.

Integration of the signals yielded the exchange or “flipping rate” of the adamantly groups within the cage at different temperatures:

T/K	k/s^{-1}
288	0.20
298	0.32
308	0.46
318	0.64

Activation parameters (298 K)

Ligand: $\Delta H^{\ddagger}$ = 64.0 kJ·mol^{-1} $\Delta S^{\ddagger}$ = 11.4 J·K^{-1}·mol^{-1} $\Delta G^{\ddagger}$ = 60.6 kJ·mol^{-1}

Cage: $\Delta H^{\ddagger}$ = 26.9 kJ·mol^{-1} $\Delta S^{\ddagger}$ = – 164.5 J·K^{-1}·mol^{-1} $\Delta G^{\ddagger}$ = 75.9 kJ·mol^{-1}

Eyring plot analysis for the flipping processes observed for ligand $\mathbf{L}^2$ and cage $[Pd_2\mathbf{L}^2_4]$:

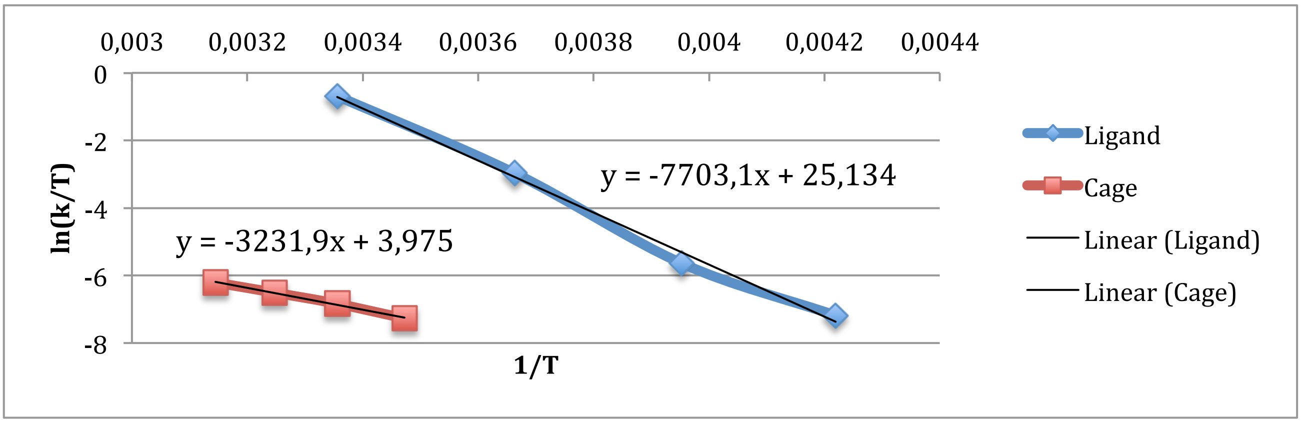

3.3 NOESY measurements of $[Pt_2L^2_4]$

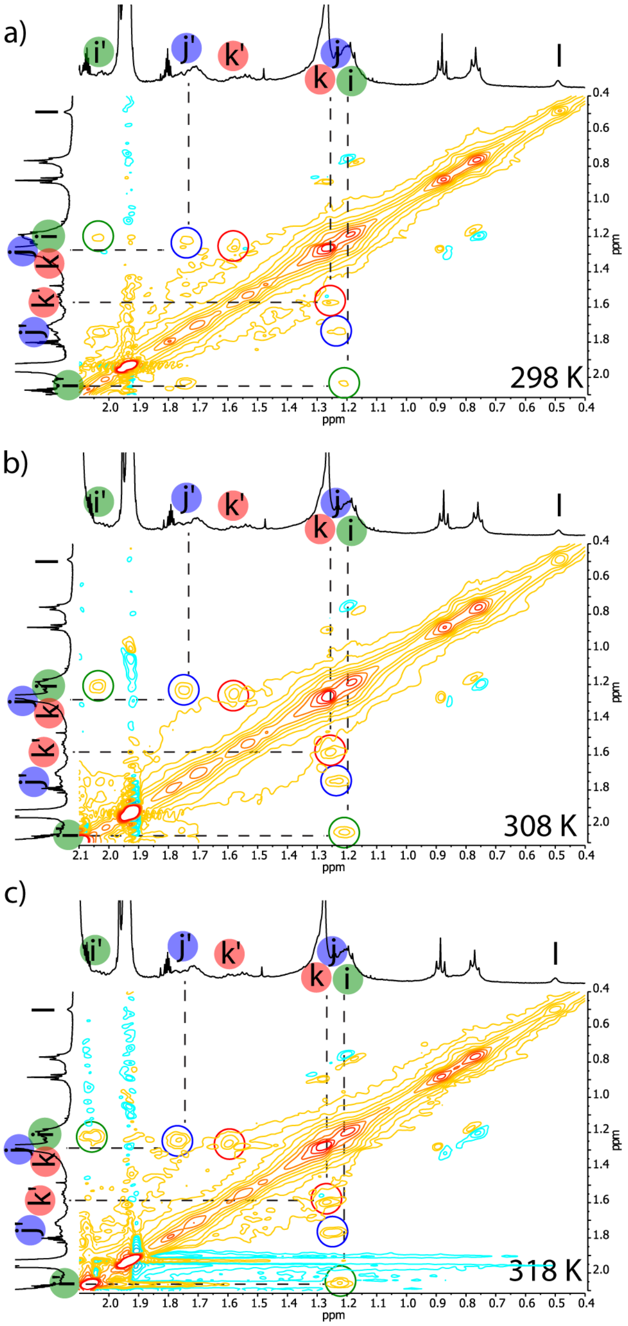

Figure SI 19 ^{1}H-^{1}H NOESY spectra of cage $[Pt_2\mathbf{L^2}_4]$ (500 MHz, CD_3CN, 0.5 s mixing time) at different temperatures: a) 298 K, b) 308 K, and c) 318 K. The intensities of the highlighted exchange signals between inward (i', j' and k') and outward (i, j, k) pointing protons are increasing with rising temperature. The rate constants could be determined through integration: 298 K: k = 0.13 s^{-1}, 308 K: k = 0.22 s^{-1} and 318 K: k = 0.35 s^{-1}.

3.4 NOESY measurements of [$\mathbf{G^1}$@$\mathbf{Pd_2L^2_4}$]

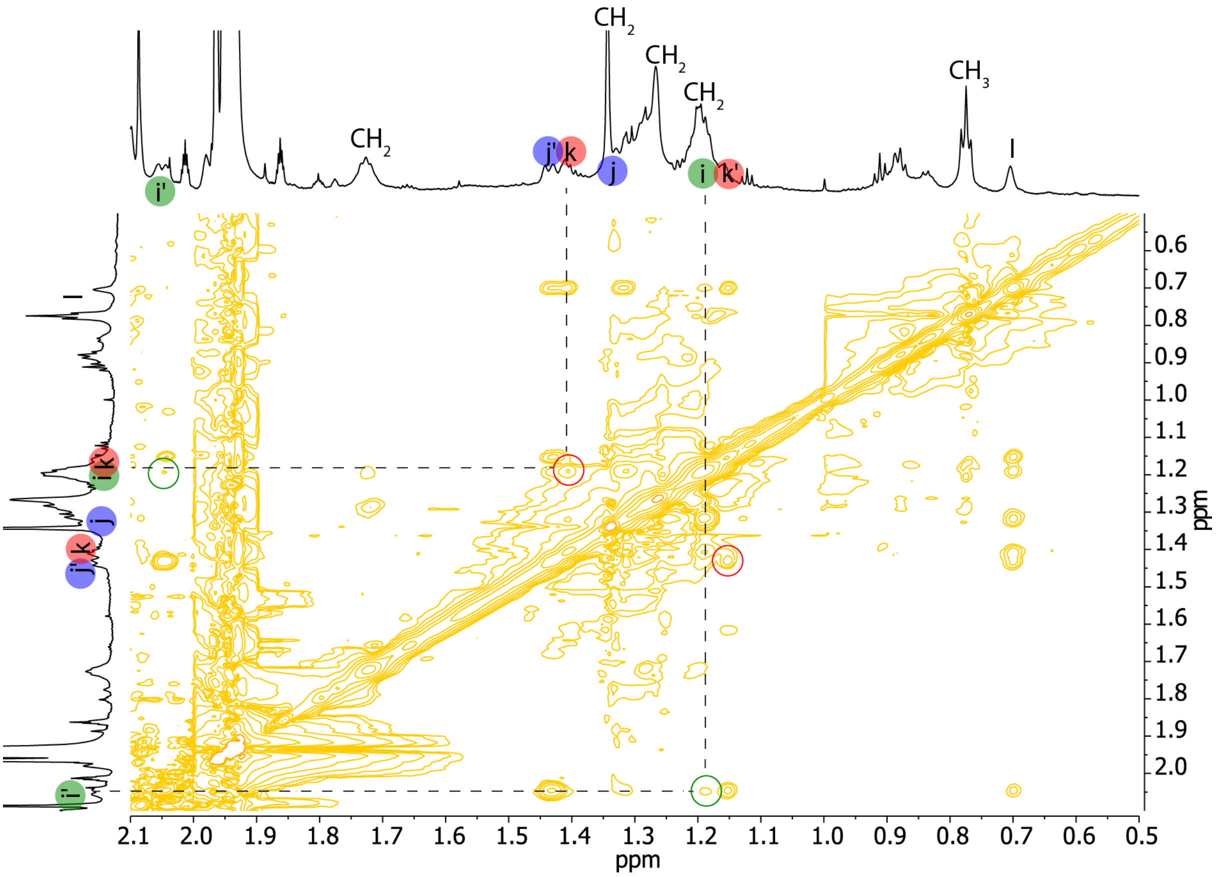

Figure SI 20 ^{1}H-^{1}H NOESY spectrum of host-guest compound [$\mathbf{G^1}$@Pd$_2$$\mathbf{L^2}_4$] (900 MHz, 298 K, CD$_3$CN, 0.5 s mixing time). The exchange signals between inside and outside pointing protons are highlighted: green = i/i' (2.05 ppm/1.19 ppm) and red = k/k' (1.42 ppm/1.17 ppm). Integration gave the exchange rate as: $k < 0.03$ s^{-1}. The exchange signals between j/j' (1.44 ppm/1.32 ppm) overlap with the diagonal baseline.

3.5 NOESY measurements of [G^2@$Pd_2L^2_4$]

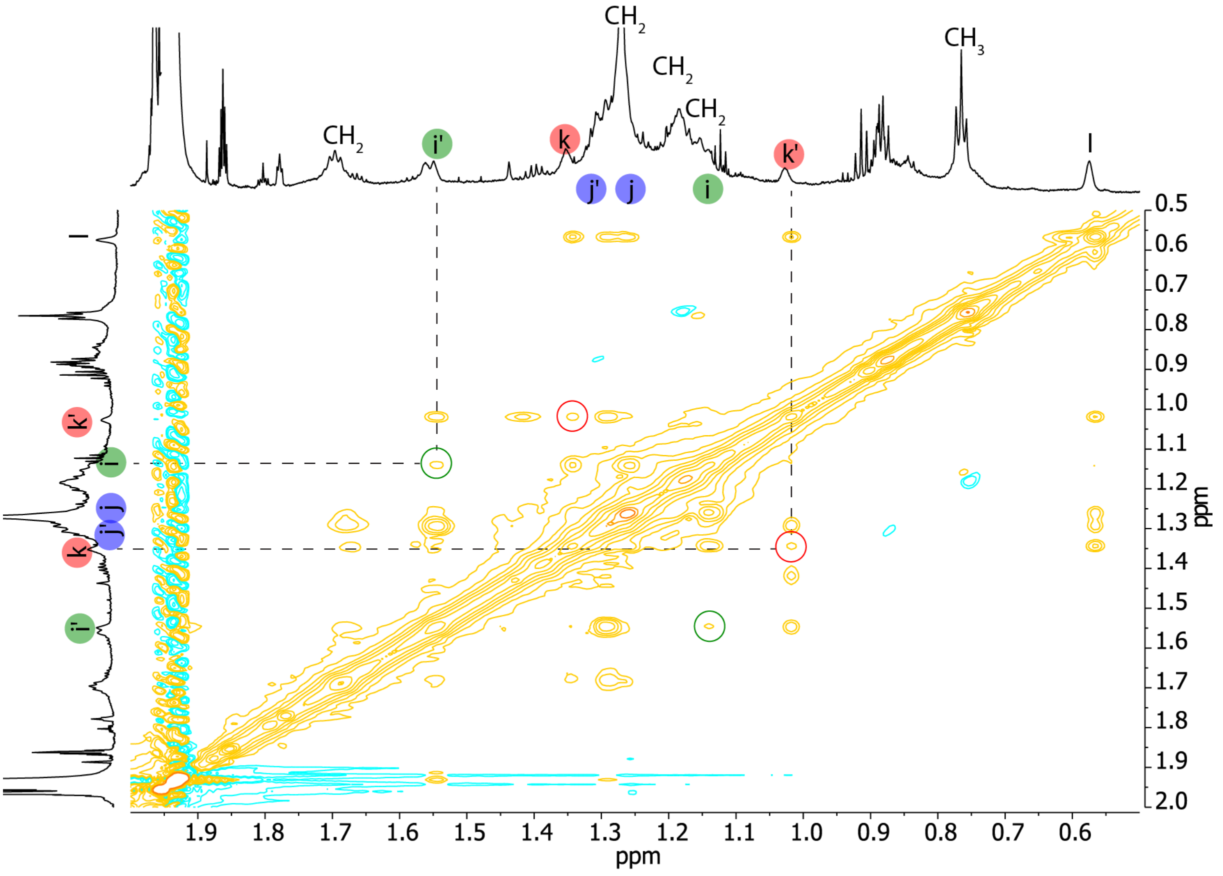

Figure SI 21 ^{1}H-^{1}H NOESY spectrum of host-guest compound [$\mathbf{G}^2$@$Pd_2\mathbf{L}^2_4$] (900 MHz, 298 K, CD_3CN, 0.5 s mixing time). Exchange signals between inside and outside pointing protons are highlighted: green = i/i' (1.54 ppm/1.14 ppm) and red = k/k' (1.35 ppm/1.02 ppm). Integration gave the exchange rate as: $k < 0.02\ s^{-1}$. The exchange signals between j/j' (1.33 ppm/1.25 ppm) are hidden in the diagonal.

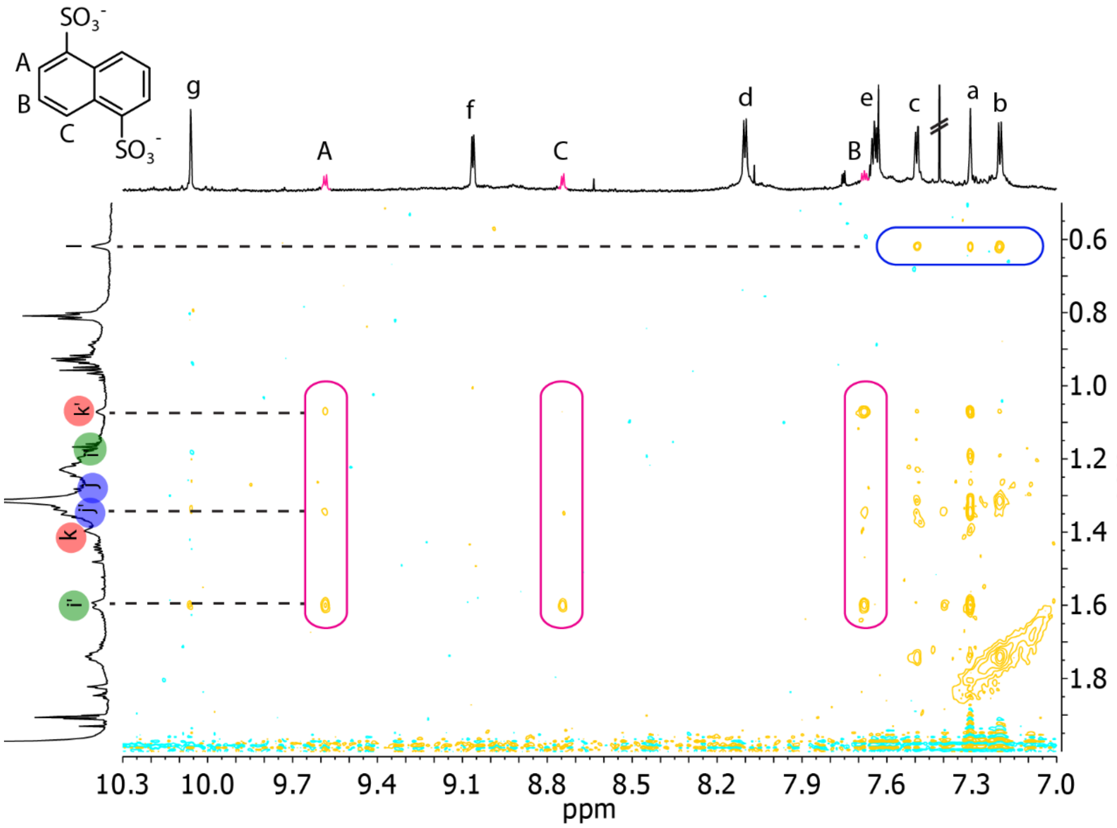

Figure SI 22 ^{1}H-^{1}H NOESY spectrum of host-guest compound [$\mathbf{G^2}$@$Pd_2\mathbf{L^2}_4$] (900 MHz, 298 K, CD_3CN). Highlighted are NOE contacts between guest $\mathbf{G^2}$ and inside pointing adamantyl protons i', j' and k' (pink) and intramolecular contacts between the ligands (blue).

3.6 EXSY Measurements

3.6.1 EXSY measurement of a 1:1 mixture of [$Pd_2L^2_4$] and [$G^1@Pd_2L^2_4$]

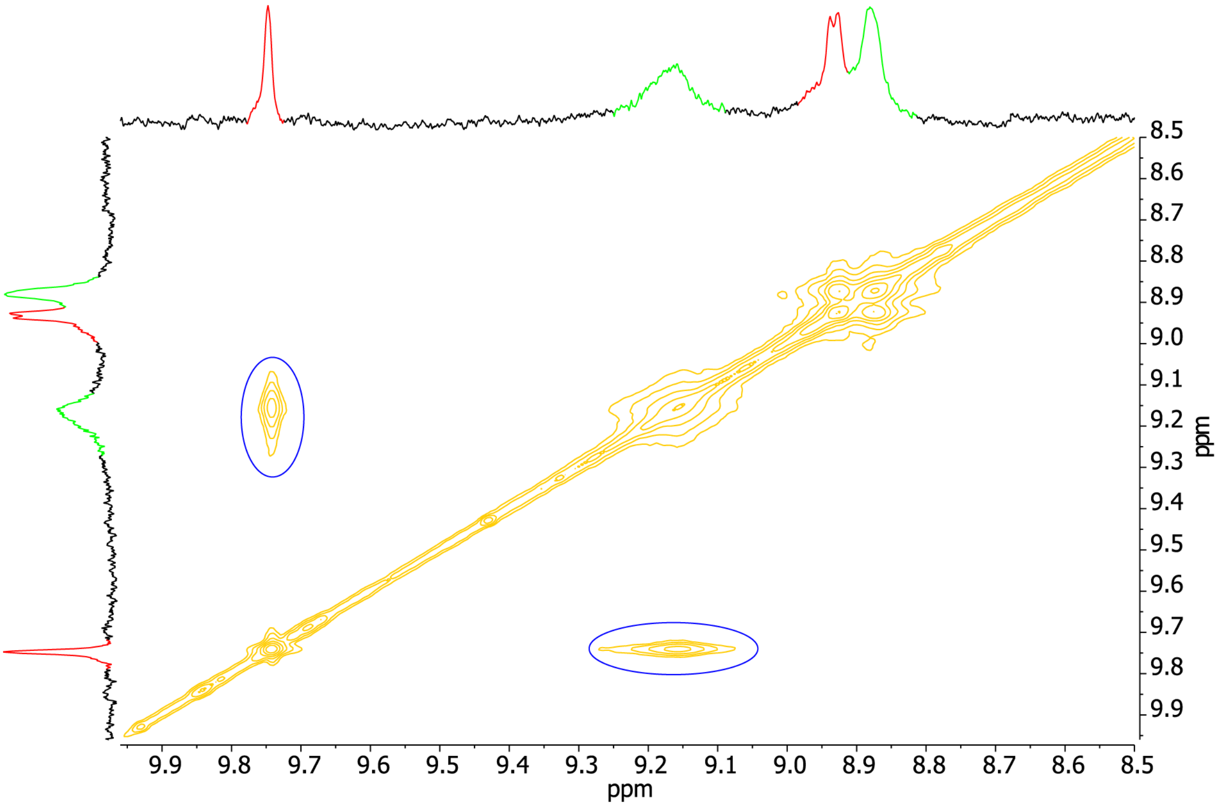

Figure SI 23 ^1H-^1H EXSY spectrum (500 MHz, 298 K, CD_3CN, 0.1 s mixing period) of a 1:1 mixture of cage [$Pd_2\mathbf{L}^{\mathbf{2}}_4$] (green) and host-guest compound [$\mathbf{G}^{\mathbf{1}}@Pd_2\mathbf{L}^{\mathbf{2}}_4$] (red). Exchange is observed between the empty cage and the host-guest complex (highlighted in blue). The rate constant could be determined *via* integration to $k_{in} = k_{out} \sim 7\ s^{-1}$ (due to signal broadening this value has an error of ~20%).

3.7 EXSY measurement of a 1:1 mixture of [$Pd_2L^2_4$] and [$G^2@Pd_2L^2_4$]

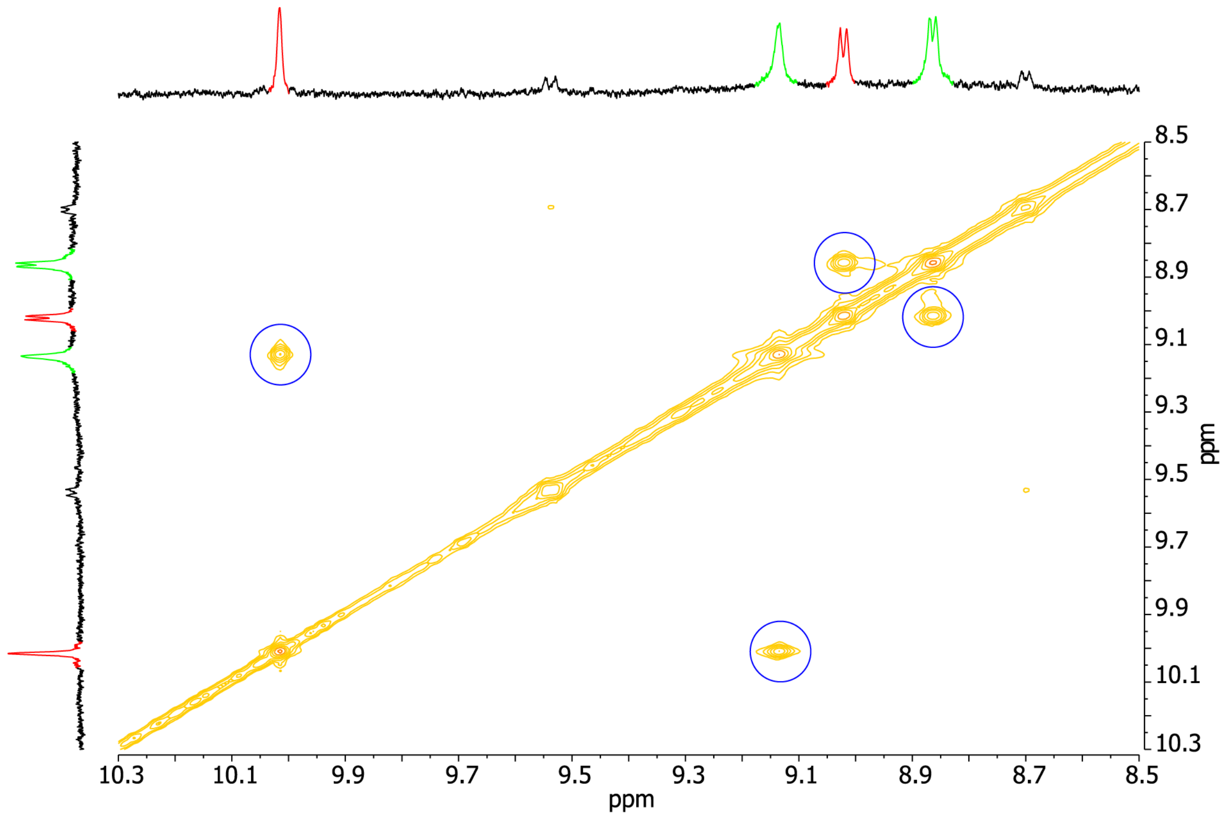

Figure SI 24 ^{1}H-^{1}H EXSY spectrum (500 MHz, 298 K, CD_3CN, 0.1 s mixing period) of a 1:1 mixture of cage [$Pd_2\mathbf{L}^2_4$] (green) and host-guest compound [$\mathbf{G}^2@Pd_2\mathbf{L}^2_4$] (red). Exchange is observed between the empty cage and the host-guest complex (highlighted in blue). The rate constant could be determined *via* integration to $k_{in} = k_{out} \sim 4\ s^{-1}$.

3.7.1 EXYS measurement of a mixture of ligand L[2] and [Pd$_2$L$^2_4$] cage

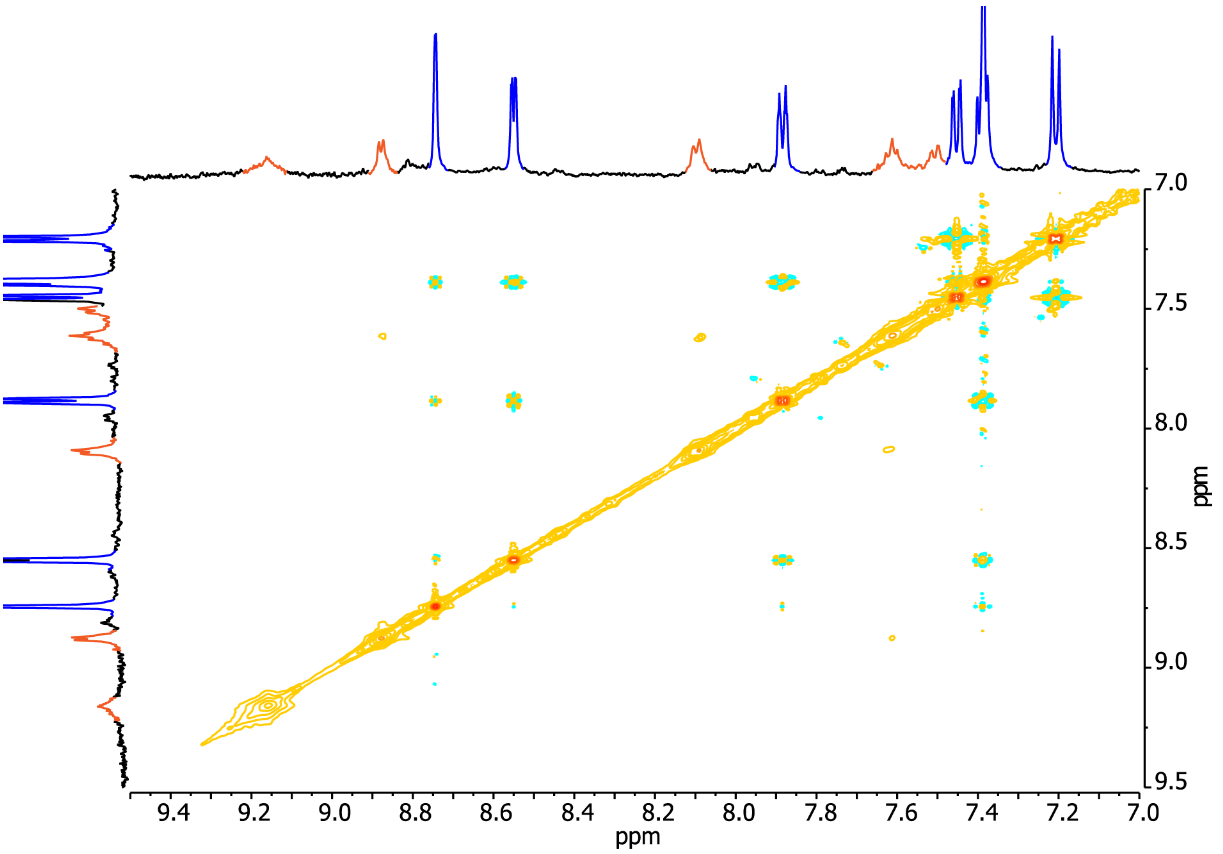

Figure SI 25 ^{1}H-^{1}H EXSY spectrum (500 MHz, 298 K, CD$_3$CN, 0.1 s mixing period) of a 3:1 mixture of ligand **L^2** (blue) and cage [Pd$_2$**L^2**$_4$] (orange) at room temperature, illustrating that there is no exchange between free ligand and the cage within the mixing time.

3.8 Addition of pyridine to $[Pd_2L^2_4]$

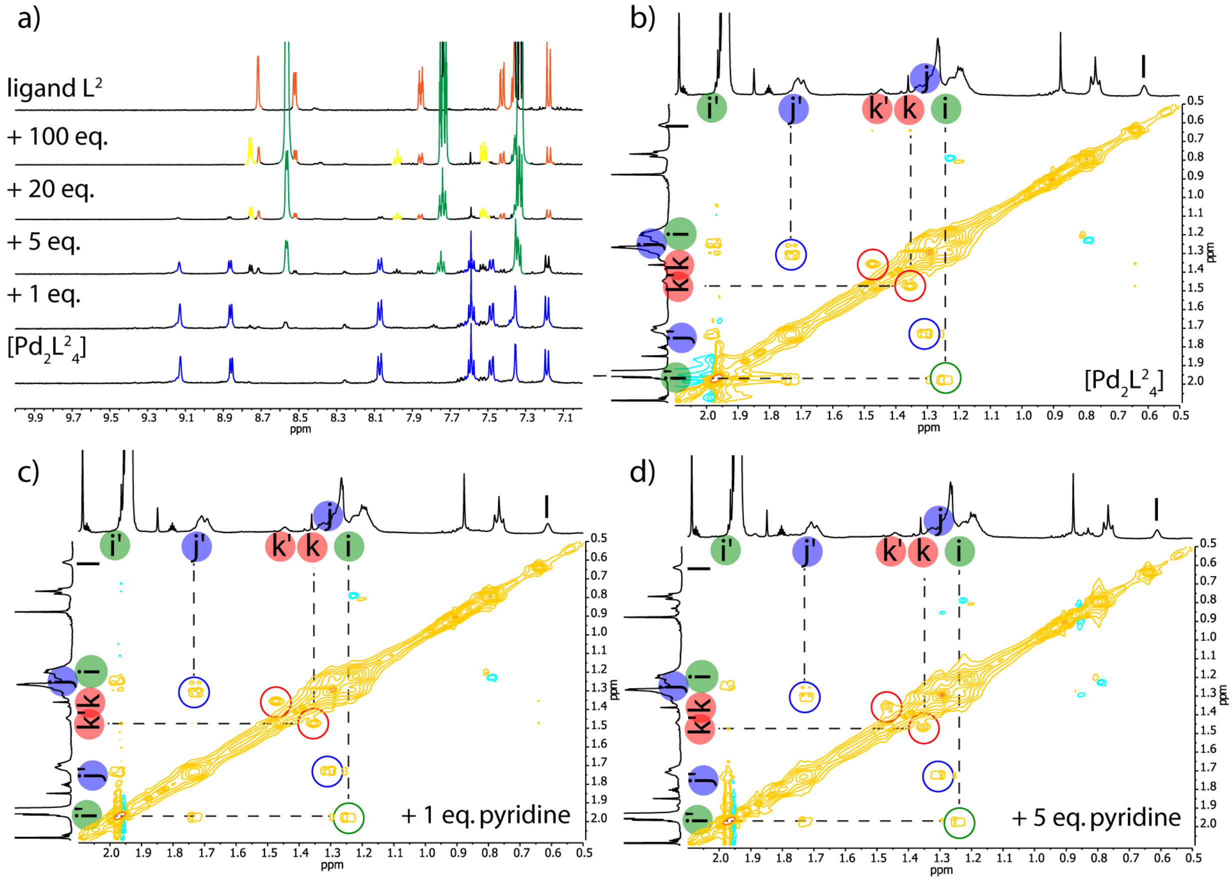

Figure SI 26 a) ^{1}H NMR titration (500 MHz, 298 K, CD_3CN) with 1 eq., 5 eq, 20 eq. and 100 eq. of pyridine to the $[Pd_2\mathbf{L^2}_4]$ cage. After 20 eq. of pyridine the cage is disassembled to free ligand and $Pd(pyridine)_4$. Color code = <u>blue</u>: $[Pd_2\mathbf{L^2}_4]$, <u>green:</u> free pyridine, <u>orange</u>: free ligand $\mathbf{L^2}$, <u>yellow</u>: $Pd(pyridine)_4$. b–d) ^{1}H-^{1}H NOESY spectra (500 MHz, 298 K, CD_3CN, 0.5 s mixing period) of the $[Pd_2\mathbf{L^2}_4]$ cage (b), with 1 eq. (c), or 5 eq. pyridine (d). The intensity of the highlighted exchange signals between inward (i', j' and k') and outward (i, j, k) pointing protons do not change with increasing pyridine concentration, indicating pyridine is not acting as a competitive ligand and has no influence on the exchange rate of the adamantyl group. The exchange rate could be determined as 0.32 s^{-1} for $[Pd_2\mathbf{L^2}_4]$. Addition of 1 eq. pyridine gave an exchange rate of 0.25 s^{-1}, which did not change after addition of 5.0 eq. pyridine.

4 Cyclic Voltammetry

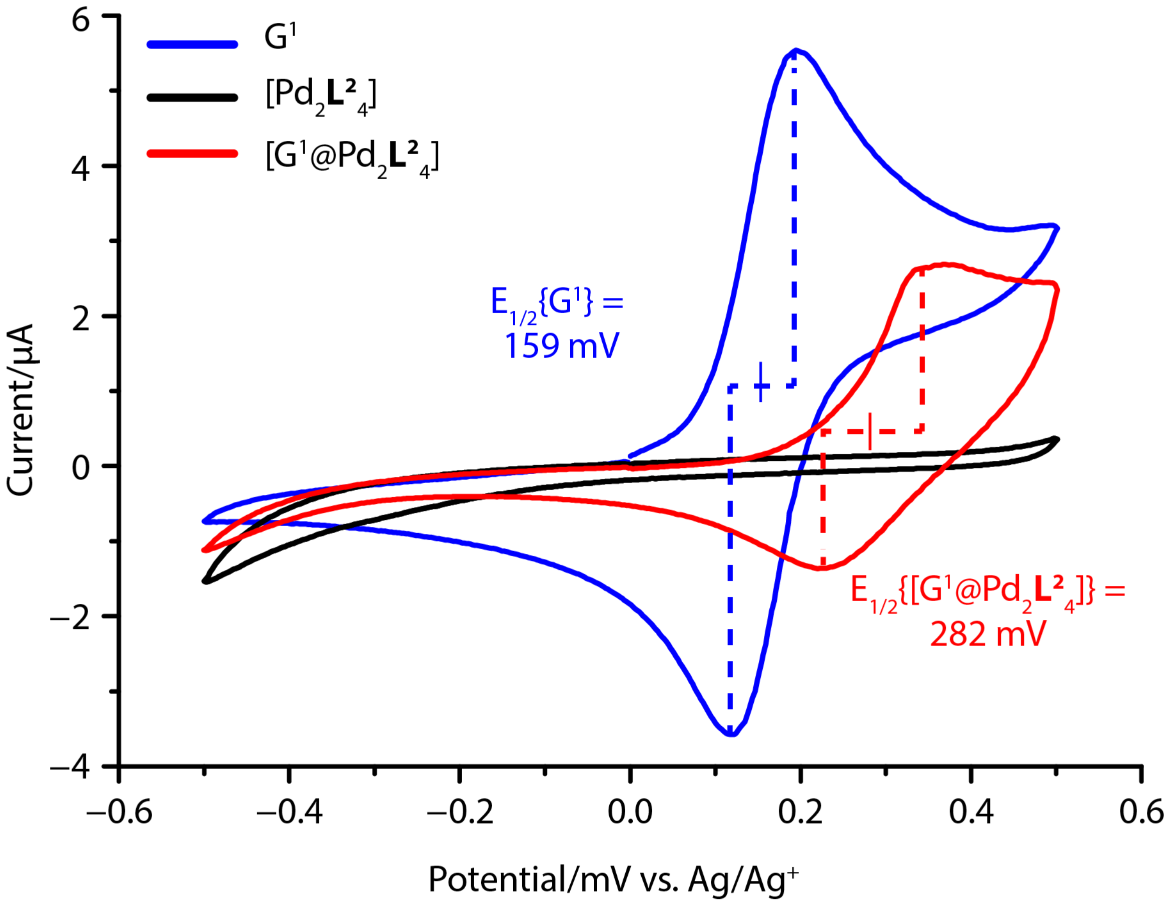

Figure SI 27 Cyclic voltammograms of the free guest **G**[1] (blue), coordination cage [Pd_2**L**$^{\mathbf{2}}_4$] (black) and host-guest complex [**G**$^{\mathbf{1}}$@Pd_2**L**$^{\mathbf{2}}_4$] (red) at 298 K, 0.07 mM, 0.1 M NBu_4PF_6, scan rate 0.1 Vs^{-1} (potentials given against an Ag/$AgNO_3$ reference electrode; [$AgNO_3$] = 0.001 M). While the cage [Pd_2**L**$^{\mathbf{2}}_4$] does not show a redox reaction in the examined potential range, the encapsulated guest **G**$^{\mathbf{1}}$ shows an anodic shift of 123 mV for $E_{1/2}$($Fe^{II/III}$) with respect to free guest **G**$^{\mathbf{1}}$. Inside the cationic coordination cage [Pd_2**L**$^{\mathbf{2}}_4$] it becomes more difficult to oxidize the guest.

5 X-Ray Data

Single crystals suitable for X-ray structural analysis were mounted at room temperature in Paratone N inert oil. Single crystal X-ray diffraction data were collected at the Swiss Light Source at beamline PXII. The data were integrated with XDS[5] and converted with the utility program xds2sad by G. M. Sheldrick. An empirical absorption correction with SADABS[6] was applied and the structures were solved by direct methods.[7] The structure models were refined against all data by full-matrix least-squares methods on F^2 with the program shelxl2014.[8] Non-hydrogen-atoms (with the exception of a disordered ligand on an inversion center in structure [**G**$^{\mathbf{5}}$@$Pd_2L^2_4$] and several atoms in highly disordered hexyl groups) were refined with anisotropic displacement parameters. The hydrogen atoms were refined isotropically on calculated positions using a riding model with U_{iso} values constrained to 1.2/1.5 U_{eq} of their parent atoms. The SQUEEZE method provided by the program Platon was used to improve the contrast of the electron density map of the structures [Pd_2**L**$^{\mathbf{2}}_4$], [**G**$^{\mathbf{4}}$@Pd_2**L**$^{\mathbf{2}}_4$] and [**G**$^{\mathbf{5}}$@Pd_2**L**$^{\mathbf{2}}_4$].

Table SI_1 Crystal data and structure refinement.

Structure	[$\mathbf{G}^1$@$Pd_2\mathbf{L}^2_4$]	[$Pd_2\mathbf{L}^2_4$]	[$\mathbf{G}^4$@$Pd_2\mathbf{L}^2_4$]	[$\mathbf{G}^5$@$Pd_2\mathbf{L}^2_4$]
CCDC number	1053082	1053083	1053081	1053080
Empirical formula	$C_{192}H_{177}Fe_2N_{12}O_{15}Pd_2S_4$	$C_{172}H_{164}B_4F_{16}N_{12}Pd_2$	$C_{185}H_{173}B_2F_8N_{14}O_6Pd_2S_2$	$C_{177}H_{153}B_2F_8N_{12}O_8Pd_2S_2$
Formula weight	3345.19 g mol^{-1}	2959.18 g mol^{-1}	3138.90 g mol^{-1}	3021.04 g mol^{-1}
Temperature	100(2) K	100(2) K	100(2) K	100(2) K
Wavelength	1.000 Å	1.000 Å	0.97896 Å	0.97896 Å
Crystal system	Triclinic	Triclinic	Triclinic	Triclinic
Space group	*P-1*	*P-1*	*P-1*	*P-1*
Unit cell dimensions	a = 18.000(4) Å<br>b = 24.665(5) Å<br>c = 25.221(5) Å<br>Alpha = 109.16°<br>Beta = 97.62°<br>Gamma = 100.30°	a = 16.196(3) Å<br>b = 16.375(3) Å<br>c = 17.065(3) Å<br>Alpha = 74.20°<br>Beta = 62.65°<br>Gamma = 71.79°	A = 15.190(3) Å<br>B = 18.168(4) Å<br>C = 18.770(4) Å<br>Alpha = 64.90°<br>Beta = 79.12°<br>Gamma = 76.17°	A = 19.202(4) Å<br>B = 20.734(4) Å<br>C = 23.321(5) Å<br>Alpha = 93.96°<br>Beta = 108.19°<br>Gamma = 92.25°
Volume	10184(4) Å3	3774.0(18) Å3	4532(2) Å3	9090(4) Å3
Z	2	1	1	2
Density (calculated)	1.091 Mg/m^3	1.302 Mg/m^3	1.150 Mg/m^3	1.104 Mg/m^3
Absorption coefficient	1.018 mm^{-1}	0.766 mm^{-1}	0.657 mm^{-1}	0.652 mm^{-1}
F(000)	3482	1536	1635	3133
Crystal size	0.011 x 0.011 x 0.005 mm^3	0.010 x 0.010 x 0.005 mm^3	0.011 x 0.011 x 0.005 mm^3	0.011 x 0.011 x 0.005 mm^3
Theta range for data collection	1.229 to 27.136°	1.864 to 27.274°	1.658 to 29.356°	1.221 to 24.196°
Index range	-14<=h<=13, -21<=k<=21, -22<=l<=22	-14<=h<=13, -13<=k<=13, -14<=l<=14	-14<=h<=14, -18<=k<=18, -18<=l<=18	-15<=h<=15, -17<=k<=76, -18<=l<=17
Reflections collected	44258	14035	36786	32793
Independent reflections	13349<br>[R(int) = 0.0927]	4035<br>[R(int) = 0.1575]	8052<br>[R(int) = 0.0908]	9379<br>[R(int) = 0.0483]
Completeness to theta	82.2 % [27.163°]	67.1 % [27.274°]	84.4 % [29.356°]	83.9 % [24.196°]
Refinement method	Full-matrix least-squares on F2	Full-matrix least-squares on F2	Full-matrix least-squares on F2	Full-matrix least-squares on F2
Data / restraints / parameters	13349 / 5905 / 2050	4082 / 1894 / 928	8052 / 3335 / 1389	9379 / 5839 / 1906
Goodness-of-fit on F2	1.162	1.280	1.086	1.388
Final R indices [I>2sigma(I)]	R1 = 0.1178<br>wR2 = 0.2941	R1 = 0.1421<br>wR2 = 0.3421	R1 = 0.0798<br>wR2 = 0.2269	R1 = 0.1071<br>wR2 = 0.3096
R indices (all data)	R1 = 0.1380<br>wR2 = 0.3132	R1 = 0.1830<br>wR2 = 0.3767	R1 = 0.0947<br>wR2 = 0.2374	R1 = 0.1254<br>wR2 = 0.3285
Largest diff. peak and hole	1.632 and<br>-0.532 e.Å-3	0.996 and<br>-0.485 e.Å-3	0.576 and<br>-0.517 e.Å-3	0.677 and<br>-0.431 e.Å-3

Further X-ray views:

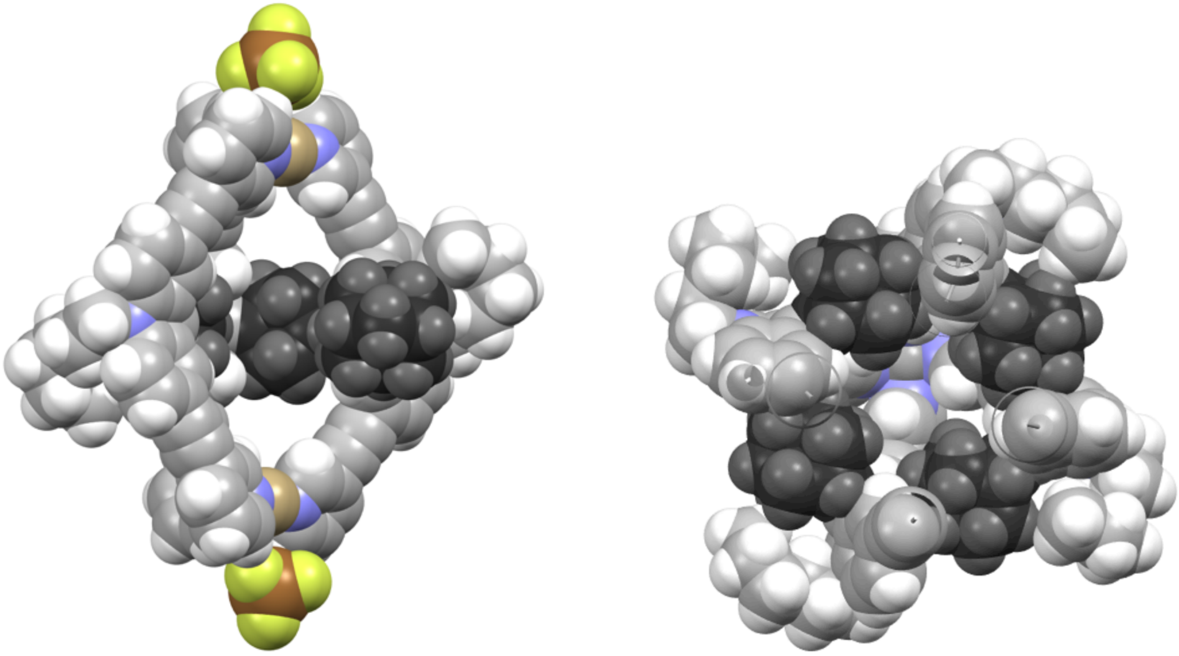

Figure SI 28 Partially clipped space-filling representations of $[Pd_2\mathbf{L}^2_4]$ from the side (left; one ligand removed) and the top (right). The adamantyl groups are highlighted in dark grey and the internal counter anions have been removed.

6 Hirshfeld surface analysis

A Hirshfeld surface analysis was performed for the host-guest complexes $[\mathbf{G}^1@Pd_2\mathbf{L}^2_4]$ and $[\mathbf{G}^5@Pd_2\mathbf{L}^2_4]$ using the software Crystal Explorer 3.1.[9] Since a few disordered solvent molecules could not be refined in the X-ray structures and the corresponding areas of residual electron density were treated by the SQUEEZE method (see above), small parts of the calculated Hirshfeld surfaces ‘bleed’ into void spaces that appear to be empty but should be filled by solvent. Nevertheless, we anticipate that the errors in the interpretations of the Hirshfeld surfaces caused by this effect are negligible since the guest molecules’ surroundings are by far dominated by the closely packed cage structure.

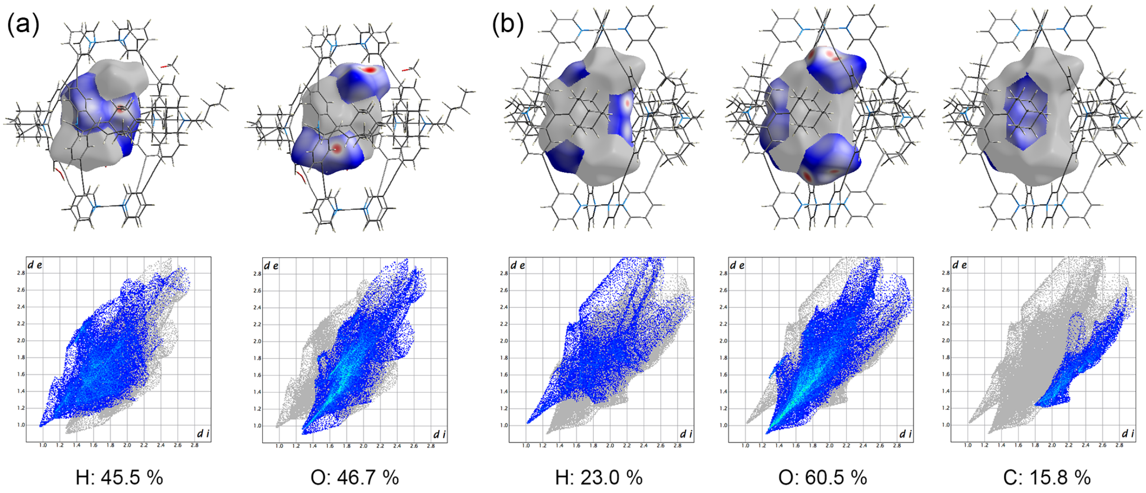

Figure SI 29 Element-mapped Hirshfeld surfaces and fingerprints of (a) $[\mathbf{G}^1@Pd_2\mathbf{L}^2_4]$ and (b) $[\mathbf{G}^5@Pd_2\mathbf{L}^2_4]$.

7 Computational details

7.1 Ligand flipping

The flipping barrier for the free ligand was computed by optimizing a connected reaction path from one orientation towards its reflection at the BP86-D3/def2-SVP (BJ damping) level of theory.[10] The computations show that the flipping occurs by traversing an extremely shallow plateau, built from two symmetric transition states. Due to the flatness of the potential energy surface in this region, we were unable to localize a minimum in between. Hence, the structure of the transition state depicted in Fig. 4a is not as highly symmetric as one might expect. Nevertheless, the barrier should be defined by the energy difference between the found transition states and the starting structure. Free energies were computed for 298.15 K at the B3LYP-D3/def2-TZVP (BJ damping) level of theory, [10e, 11] including solvent corrections through the COSMO model (for acetonitrile).[12] The value obtained was 56.6 kJ·mol^{-1}, in close agreement with the NMR derived flipping rate constant. All calculations were carried out with the ORCA 3.0 program package.[13]

7.2 Guest binding

In order to obtain further insight into the binding of the guest molecules, in particular the role of dispersion forces, electronic structure calculations were carried out for [$Pd_2\mathbf{L}^2_4$], [$\mathbf{G}^4$@$Pd_2\mathbf{L}^2_4$] and [$\mathbf{G}^5$@$Pd_2\mathbf{L}^2_4$]. Due to the large size of these system, the calculations involving the binding pocket were limited to the four adamantane moieties (with the covalent double bond to the ligand backbone cut and capped with two hydrogen atoms). The adamantane fragments and guest compounds were optimized at the B3LYP-D3/def2-TZVP level of theory (with Becke-Johnson type damping), and then overlapped with the crystal structure positions. These should correspond to a suitable average conformation of the ligands and guest molecules. Also, given the small space available in the pocket, dynamic fluctuations in the structure should not affect our results severely. On the basis of the structures obtained, spin-component-scaled local second-order Møller-Plesset perturbation theory SCS-LMP2[14,15] calculations were performed, together with the aug'-cc-pVTZ basis set (diffuse functions on all atoms, except hydrogens).[16] Density fitting approximations were used throughout, with the default fitting basis for aug-cc-pVTZ. The latter calculations were used to investigate the interaction between the guests and the adamantane moieties. The reasons behind the choice of SCS-LMP2 are manifold. The use of local correlation reduces basis set superposition effects, which are quite significant in such host-guest binding studies. Through the use of SCS, the known overestimation of dispersion forces in MP2 theory is avoided. Finally, local correlation allows for an intuitive and direct separation of the different components of the correlation energy,[17] including dispersion, which is expected to play a major role in the stabilization of these systems. All interaction energies are solely electronic. The total energies are given according to the supermolecular (not 'supramolecular') approach, with fixed host and guest structures. The dispersion energies were obtained through the decomposition of the scaled LMP2 pair energies. Calculations in the system [$Pd_2\mathbf{L}^2_4$] were primarily intended to estimate the stabilization of the cage brought forth by dispersion interactions between the adamantane moieties in the pocket. This system was chosen since it contains the smallest guest (BF_4^-), allowing for the adamantane moieties to

come closest. In the calculations of the host-guest complexes, only the interactions of the guests with the adamantanes were considered, disregarding the interaction with the remaining parts of the ligands and the Pd ions. The latter would be particularly difficult to estimate given that the electrostatics will be strongly influenced by the solvent model used. The remaining parts of the ligands should have no strong interaction with the guests considered. All local correlation calculations were carried out with the Molpro2012.1 program package.[18]

7.3 Dispersion Interaction Density plots

In order to obtain a visual description of dispersion forces, a decomposition of the SCS-LMP2 pair energies has been carried out. The energy terms associated with a double excitation i->a and j->b, with i/a being occupied/virtual orbitals located in monomer A , j/b occupied/virtual orbitals in monomer B, were summed together into $\varepsilon^{d}_{(ij)}$ dispersion energy contributions. These are used to compute the dispersion interaction density matrix D for an arbitrary monomer A as:

$$D^{A}_{\mu\nu} = \sum_{i \in A} (\sum_{B} \sum_{j \in B} \varepsilon^{d}_{(ij)})\, P^{i}_{\mu\nu}$$

Whereby P^{i} is the density matrix for the occupied orbital i. A numerical grid can then be generated and the DID value at a given point in space computed as

$$T(r) = \sum_{\mu\nu} D^{A}_{\mu\nu}\, \chi_{\mu}(r) \chi_{\nu}(r)$$

In the end, one obtains a tensor in cartesian space, with a value proportional to the dispersion interaction of each orbital and its density. Further details into the method will be presented in another publication.[19]

8 References

[1] Miyashita, K.; Minagawa, M., Ueda, Y., Tada, Y., Hoshino, N.;. Imanishi,T. *Tetrahedron* **2001**, *57*, 3361.
[2] Löffler, S.; Lübben, J.; Krause, L.; Stalke, D.; Dittrich, B.; Clever, G. H. *J. Am. Chem. Soc.* **2015**, *137*, 1060.
[3] a) Clever, G. H.; Kawamura, W.; Shionoya, M. *Inorg. Chem.* **2011**, *50*, 4689. ; b) Clever, G. H.; Tashiro, S.; Shionoya, M. *Angew. Chem. Int. Ed.* **2009**, *48*, 7010. b) Clever, G. H.; Tashiro, S.; Shionoya, M. *J. Am. Chem. Soc.* **2010**, *132*, 9973.
[4] Lawrence, N. S.; Gary, Tustin, G. J.; Faulkner, M.; Jones, T. G. J. *Electrochemica Acta*, **2006**, *52*, 499.
[5] Kabsch, W. *Acta Cryst*. **2010**, *D66*, 125.
[6] Sheldrick, G. M. *SADABS,* Universität Göttingen, Germany, **2000**.
[7] Schneider, T. R.; Sheldrick, G. M. *Acta Cryst*. **2002**, *D58*, 1772.
[8] Sheldrick, G. M. *Acta Crystallogr. Sect. A* **2008**, *64*, 112.
[9] Spackman, M. A.; Jayatilaka, D. *Cryst. Eng. Comm.* **2009**, *11*, 19.
[10] a) Becke, A. D. *Phys. Rev. A* **1988**, *38*, 3098. b) Perdew, J. P. *Phys. Rev. B* **1986**, *33*, 8822. c) Grimme, S.; Ehrlich, S.; Goerigk, L. *J. Comput. Chem.* **2011**, *32*, 1456. d) Grimme S.; Antony, J.; Ehrlich, S.; Krieg, H. *J. Chem. Phys.* **2010**, *132*, 154104. e) Weigend, F.; Ahlrichs, R. *Phys. Chem. Chem. Phys.* **2005**, *7*, 3297.
[11] Becke, A. D. *J. Chem. Phys.* **1993**, *98*, 5648.
[12] Klamt, A.; Schüürmann, G. *J. Chem. Soc., Perkin Trans. 2* **1993**, *5*, 799.
[13] Neese, F. *WIREs Comput. Mol. Sci.* **2012**, 2, 73.
[14] Grimme, S. *J. Chem. Phys* **2003**, *118.20*, 9095.
[15] Werner, H.-J.; Manby, F. R.; Knowles, P. J. *J. Chem. Phys.* **2003**, *118*, 8149.
[16] a) Dunning, Jr., T. H. *J. Chem. Phys.* **1989**, *90*, 1007. b) Kendall, R. A.; Dunning, Jr., T. H.; Harrison, R. J. *J. Chem. Phys.* **1992**, *96*, 6796.
[17] Schütz, M.; Rauhut, G.; Werner, H.-J. *J. Phys. Chem. A* **1998**, *102*, 5997.
[18] Werner, H.-J.; Knowles, P. J.; Knizia, G.; Manby, F. R.; Schütz M. *Molpro*, version 2012.1, a package of *ab initio* programs, **2012**, see http://www.molpro.net.
[19] Wuttke, A.; Andrejić, M; Mata, R. A. *to be submitted*.
